# The shared neurobiological basis of developmental dyslexia and developmental stuttering: A meta-analysis of functional and structural MRI studies

**DOI:** 10.1016/j.ijchp.2024.100519

**Published:** 2024-11-10

**Authors:** Huan Ren, Yi zhen Li, Hong-Yan Bi, Yang Yang

**Affiliations:** a Key Laboratory of Behavioral Science, Center for Brain Science and Learning Difficulties, Institute of Psychology, Chinese Academy of Sciences, Beijing, 100101, China; b Department of Psychology, University of Chinese Academy of Sciences, Beijing 100049, China; c Center for Language and Brain, Shenzhen Institute of Neuroscience, Shenzhen 518057, China

**Keywords:** Developmental dyslexia, Persistent developmental stuttering, Functional magnetic resonance imaging, Voxel-based morphometry, Meta-analysis

## Abstract

**Background:**

Developmental dyslexia (DD) and persistent developmental stuttering (PDS) are the most representative written and spoken language disorders, respectively, and both significantly hinder life success. Although widespread brain alterations are evident in both DD and PDS, it remains unclear to what extent these two language disorders share common neural substrates.

**Methods:**

A systematic review and meta-analysis of task-based functional magnetic resonance imaging (fMRI) and voxel-based morphometry (VBM) studies of PDS and DD were conducted to explore the shared functional and anatomical alterations across these disorders.

**Results:**

The results of fMRI studies indicated shared hypoactivation in the left inferior temporal gyrus and inferior parietal gyrus across PDS and DD compared to healthy controls. When examined separately for children and adults, we found that child participants exhibited reduced activation in the left inferior temporal gyrus, inferior parietal gyrus, precentral gyrus, middle temporal gyrus, and inferior frontal gyrus, possibly reflecting the universal causes of written and spoken language disorders. In contrast, adult participants exhibited hyperactivation in the right precentral gyrus and left cingulate motor cortex, possibly reflecting common compensatory mechanisms. Anatomically, the analysis of VBM studies revealed decreased gray matter volume in the left inferior frontal gyrus across DD and PDS, which was exclusively observed in children. Finally, meta-analytic connectivity modeling and brain-behavior correlation analyses were conducted to explore functional connectivity patterns and related cognitive functions of the brain regions commonly involved in DD and PDS.

**Conclusions:**

This study identified concordances in brain abnormalities across DD and PDS, suggesting common neural substrates for written and spoken language disorders and providing new insights into the transdiagnostic neural signatures of language disorders.

## Introduction

Language is fundamental to success in modern literary society. However, some individuals suffer from various language disorders. Persistent developmental stuttering (PDS) and developmental dyslexia (DD) are two prevalent neurodevelopmental disorders of language that have been highly studied. PDS is a typical spoken language disorder characterized by involuntary repetitions and prolongations of syllables, particularly during connected speech (Brown et al., 2005; Büchel & Sommer, 2004; Tager-Flusberg & Cooper, 1999). In contrast, DD is a representative written language disorder characterized by difficulties in acquiring proficient reading and spelling skills, despite adequate instruction, intelligence and intact sensory abilities (Goswami, 2015; Lyon et al., 2003; Peterson & Pennington, 2012). Despite extensive investigation, the etiology of PDS and DD remains unclear.

Magnetic resonance imaging (MRI) serves as a powerful neuroimaging tool for investigating the neural correlates of language disorders. Over the past decades, functional MRI (fMRI) and structural MRI (sMRI) studies have revealed abnormalities in brain function and structure associated with various types of language disorders. With respect to PDS, task-based MRI studies have demonstrated functional disruption within multiple language and motor regions when performing speech and non-speech tasks, involving the left primary motor and premotor cortex, inferior frontal gyrus (IFG), middle frontal gyrus (MFG), precentral gyrus/postcentral gyrus (preCG/postCG), left supplementary motor area (SMA), bilateral superior temporal gyrus (STG) and middle temporal gyrus (MTG), basal ganglia, cingulate motor area, and the cerebellum (Brown et al., 2005; Garnett et al., 2019, 2018; Liu et al., 2014; Shojaeilangari et al., 2021; Watkins et al., 2008; Yang, 2009; Yang et al., 2016). For individuals with DD, numerous task-based fMRI studies have demonstrated atypical functional activation when performing linguistic and non-linguistic tasks in the left IFG, MFG, PreCG/PostCG, inferior parietal lobule (IPL), MTG, fusiform gyrus, cingulate cortex, and cerebellum (Hancock et al., 2017; Li & Bi, 2022; Richlan et al., 2011, 2010; Yan et al., 2021; Yang et al., 2022).

Voxel-based morphometry (VBM) is a frequently used method for examining the anatomical alterations associated with different language-related disorders (Jovicich et al., 2013). With respect to PDS, prior VBM studies have identified altered gray matter volume (GMV) in the left IFG and bilateral temporal regions (Beal et al., 2013; Chang et al., 2008). For individuals with DD, prior studies have demonstrated the alterations of GMV in the IFG, supramarginal gyrus (SMG), superior temporal gyrus/sulcus, inferior temporal gyrus (ITG) and cerebellum (Eckert et al., 2016; Linkersdoerfer et al., 2012; Ramus et al., 2018; Richlan et al., 2013). Overall, the widespread brain alterations identified in PDS and DD highlight the complex etiology of these language-related disorders.

These neuroimaging studies discussed above have delineated a complex map of functional and structural abnormalities in the brain associated with DD and PDS. However, a fundamental question that remains unresolved is to what extent there are shared brain markers underlying these two language disorders. This is a critical inquiry, as it deepens our understanding of the neurobiological underpinnings of both DD and PDS. Specifically, by identifying the shared brain markers of PDS and DD, we can uncover intrinsic and stable brain mechanisms that underlie these language-related conditions, irrespective of the diverse manifestations of their phenotypes. Furthermore, investigating the shared neural basis has the potential to become a key target for future diagnostic efforts and therapeutic interventions for these distinct language disorders.

Despite their categorization into distinct groups, theoretical hypotheses and empirical findings suggest the existence of a common neural mechanism underlying these two disorders (Algaidi et al., 2023; Ardila et al., 1994; Elsherif et al., 2021). From a theoretical perspective, the procedural deficit hypothesis offers an explanatory framework for explaining the cause of various language disorders (Ullman et al., 2020; Ullman & Pierpont, 2005). According to this hypothesis, different types of language disorders may share procedural processing abnormalities, specifically involving deficits in functions that depend on cortico-basal ganglion-thalamocortical circuits (Krishnan et al., 2016; Ullman et al., 2020). The procedural deficit hypothesis is further supported by the findings of a recent meta-analysis, which elucidate the basal ganglia as the neuroanatomical signature of developmental language disorder (Ullman et al., 2024). Additionally, the phonological deficit hypothesis can account for both DD and PDS. Phonological deficits have been established as the core factor of DD (Bishop & Snowling, 2004; Catts et al., 2005; Ramus, 2003; Ramus et al., 2013; Snowling, 2001). Similarly, phonological impairments are also observed in individuals with PDS, such as the developmental shift in phonological encoding from holistic to incremental processing (Byrd et al., 2007), and reduced phonological memory (Elsherif et al., 2021). These phonological deficits may be attributable to functional and structural abnormalities in the auditory cortex, as revealed in both DD (Gertsovski & Ahissar, 2022; Jaffe-Dax et al., 2018; Kuhl et al., 2020) and PDS (Beal et al., 2010; Connally et al., 2018). Second, some fMRI studies on healthy individuals discovered common neural activity for processing spoken and written languages, engaging distributed brain regions within a frontal-parietal-temporal language network, including the IFG (Broca's area) (Sahin et al., 2009), and the left occipitotemporal areas (visual word form area, VWFA) (Longcamp et al., 2019; Qin et al., 2021). Additionally, a growing body of genetic studies has pinpointed a common genetic architecture contributing to susceptibility to both spoken and written language impairments, including genes such as forkhead box P2 (FOXP2), contactin-associated protein-like 2 (CNTNAP2), and C-MAF inducing protein (CMIP) (Graham & Fisher, 2013; Newbury et al., 2010; Paracchini, 2011; Whitehouse et al., 2011). These shared genetic mutations may modulate the alterations of core brain circuits of language development. However, developmental and environmental variables are likely to interact with genetic factors to shape brain dysfunction, ultimately leading to different phenotypes of language disorders. Finally, researchers have started to adopt a transdiagnostic perspective in examining neurodevelopmental and psychiatric conditions, rather than focusing solely on disorder-specific mechanisms (Astle et al., 2022; Koomar & Michaelson, 2020). Studies in psychiatric disorders have revealed common anatomical alterations across a wide range of psychiatric conditions (Goodkind et al., 2015; Wise et al., 2017), suggesting that these disorders may be underpinned by shared brain markers. It is expected that, similar to findings in the field of psychiatric research, shared brain markers may also exist across different types of language disorders.

Meta-analysis is an effective and efficient approach to synthesize results from various language disorders, allowing for quantitative exploration of shared brain signatures across different types of disorders. This strategy has been widely applied to identify common brain signatures of mental illnesses (Madeleine Goodkind et al., 2015; Wise et al., 2017) and language/speech disorders (Liégeois et al., 2014). Therefore, we conducted a meta-analysis to analyze the findings of fMRI and VBM studies on DD and PDS. First, we aggregated the two common types of language disorders into a unified disorder group to identify the shared core brain regions displaying functional or anatomical alterations. Our rationale was to uncover potential shared brain regions for language dysfunction across different age groups and language modalities. Subsequently, acknowledging the influence of neurodevelopment, we performed secondary analyses by categorizing the participants into child and adult groups. Finally, we characterized the functional connectivity profiles and cognitive significance of the shared brain regions that exhibit common functional or structural abnormalities across PDS and DD, aiming to illuminate how abnormalities in these regions contribute to these two types of language disorders. To this end, we conducted two complementary analyses using datasets from the BrainMap database (www.brainmap.org). First, we employed meta-analytic connectivity modeling (MACM), a validated method for identifying brain regions co-activated with a given seed region across multiple neuroimaging studies (Eickhoff et al., 2011; Robinson et al., 2012), to delineate large-scale functional connectivity patterns. Then, we conducted a behavioral domain analysis to roughly identify the cognitive functions (five main categories: action, cognition, emotion, interoception and perception) associated with the shared brain regions (Robinson et al., 2012).

## Methods

### Literature search and selection criteria

**Fig. 1:**
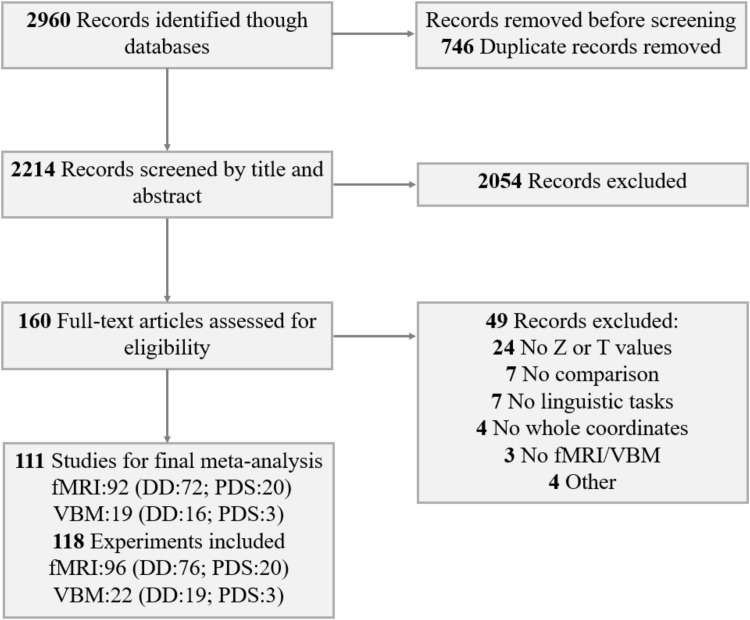
Flow diagram of study selection. **Abbreviation:** DD: developmental dyslexia; PDS: persistent developmental stuttering; fMRI: functional magnetic resonance imaging; VBM: voxel-based morphometry.

This study follows the guidelines of the Preferred Reporting Items for Systematic Reviews and Meta-Analyses (PRISMA) (Fig. 1 and eTable 1). We searched PubMed and Web of Science for fMRI and VBM studies on DD and PDS published between January 1, 1986 and March 31, 2023. The key search terms included (('dyslexia' OR 'reading disorder' OR 'reading impairment' OR 'reading difficulty' OR 'reading disability') OR ('stutter' OR 'stutterer' OR 'stuttering')) AND ('fMRI' OR 'functional magnetic resonance imaging' OR 'neuroimaging' OR 'functional MRI' OR 'functional imaging' OR 'VBM' OR 'voxel-based morphometry').

Inclusion criteria comprised studies that: (1) employed fMRI or VBM to investigate GMV in individuals with DD or PDS; (2) utilized whole-brain analysis; (3) reported group comparisons with healthy age-matched controls; (4) provided peak coordinates in Talairach space or Montreal Neurological Institute (MNI) space; (5) reported effect sizes (*t*-values and *z*-values); and (6) were peer-reviewed original papers published in English. Exclusion criteria encompassed studies that: (1) focused solely on region of interest (ROI) analysis; (2) investigated resting-state activity; (3) did not report group differences; (4) conducted direct group comparisons only between disorders and reading level control readers; (5) studied participants at risk for these disorders; (6) focused on non-linguistic tasks; (7) were case studies; (8) were meeting, review, or meta-analysis papers. In addition, we adopted the criterion of including >10 studies for each type of disorder, following recommendations for sufficient power in signed differential mapping (SDM) meta-analyses (Madeleine Goodkind et al., 2015; Radua & Mataix-Cols, 2009). Based on these criteria, 92 fMRI studies (DD: 72; PDS: 20) and 19 VBM studies (DD: 16; PDS: 3) were included in the final analysis.

### Data extraction

Two independent reviewers (H.R. and YZ. L.) evaluated the titles, abstracts, and full-text articles against the inclusion criteria, and conducted data extraction using a Microsoft Excel spreadsheet. Any conflicts or discrepencies were resolved by a third reviewer (Y.Y.).

As depicted in Fig. 1, the present meta-analysis incorporated 118 experiments (from 111 papers), encompassing 96 fMRI experiments and 22 VBM experiments (eTable 2 and eTable 3). The following information was extracted from the original publications: first author's name, publication year, writing system, number of participants, mean age of participants, fMRI task, and significance threshold. Additionally, to facilitate voxel-wise meta-analysis, peak coordinates and statistical values (*t*-values and *z*-values) were extracted.

### Meta-analysis procedure

We conducted a voxel-wise meta-analysis using SDM-PSI version 6.21 (Albajes-Eizagirre et al., 2019)(see http://www.sdmproject.com). In contrast to activation likelihood estimation (ALE) or multilevel peak kernel density analysis (MKDA), SDM reconstructs positive and negative effects within the same statistical maps, preventing a voxel from appearing in opposite directions, and thereby providing a more accurate representation of the results (Radua et al., 2012). This approach has been widely employed in previous meta-analyses (Li & Bi, 2022; Pollard et al., 2023; Ranzini et al., 2022). Pre-processing followed the default settings of SDM, using a 20 mm full-width half-maximum (FWHM) anisotropic Gaussian kernel (*α* = 1.00) and 2 mm voxel size (Radua et al., 2014). The results of the meta-analysis were thresholded at a peak height of mean effect size SDM-*Z* = 1, with an uncorrected *p-value* of 0.005 at the voxel level, and a minimum of 10 voxels at the cluster level (Radua et al., 2012), in accordance with common practices in prior meta-analyses (Li et al., 2023; Zhang et al., 2022). Funnel plots and Egger's tests were used to examine the potential publication bias of each identified peak. Asymmetry in the plots or a *p-value* < 0.05 indicates significant publication bias. The inter-study heterogeneity of each cluster was measured by the *I^2^* index, which represents the proportion of total variation due to the study heterogeneity (Higgins & Thompson, 2002). An *I^2^* value greater than 50 % typically indicates substantial heterogeneity.

For both fMRI and VBM data, the initial meta-analysis incorporated the type of language disorders as a covariate. To exclude the potential impact of language differences on the neural correlates of the two developmental disorders, especially between alphabetic languages and non-alphabetic languages (Li & Bi, 2022), we restricted analysis only to studies with participants who spoke alphabetic languages and replicated the initial meta-analysis. Next, considering neurodevelopment, we conducted separate meta-analyses for the child (under 14 years old) and adult (over 14 years old) subgroups (note that functional data for children exclusively pertain to DD; refer to eTable 2 for details).

To gain deeper insights into the functional significance of the shared brain regions across DD and PDS, we conducted two complementary analyses using a dataset of healthy participants. MACM was employed to assess the brain co-activation patterns of a seed region across numerous data-driven neuroimaging experiments (Cortese et al., 2016; Eickhoff et al., 2011; Laird et al., 2009b). The co-activation analysis utilized the BrainMap database (www.brainmap.org), and GingerALE (version 3.0.2) was used to identify regions of significant convergence. The initial step of MACM involves ensuring that all experiments in the BrainMap database contain at least one activation focus within the ROI region (Laird et al., 2009a). Then, a quantitative analysis of the foci in these retrieved experiments was performed using the ALE algorithm. The ALE algorithm tested for spatial convergence of neuroimaging findings against a null distribution of random spatial association of experiments, evaluating clusters where convergence exceeded chance expectations (Eickhoff et al., 2012, 2016; Mueller et al., 2018). Therefore, the presence of significant convergence in regions other than the highest convergence of the ROIs indicates consistent co-activation across the experiments. The *p*-values were thresholded at a cluster-level family-wise error (cFWE) of *p* = 0.05 with 1000 permutations (Eickhoff et al., 2016).

For MACM analysis, four ROIs derived from the SDM meta-analysis were extracted: the left ITG (MNI coordinates: −48, −56, −16), MTG (MNI coordinates: −56, −54, 0), inferior parietal gyrus (IPG, MNI coordinates: −56, −48, 38), and SMG (MNI coordinates: −56, −50, 30) (refer to Results 3.2 for details). All ROIs are 10 mm boxes centered at the peak MNI coordinates. The ROI of the left ITG included 179 experiments (3242 subjects, 2648 foci), the ROI of the left MTG included 82 experiments (1473 subjects, 1184 foci), the ROI of the left IPG included 75 experiments (1787 subjects, 1176 foci) and the ROI of the left SMG included 71 experiments (1592 subjects, 895 foci).

The functional properties of each ROI were further examined based on the behavioral domain metadata categories available for each neuroimaging experiment in the BrainMap database. Behavioral domains include the main categories of cognition, action, perception, emotion, and interoception, along with their related subcategories (see http://brainmap.org/scribe/ for the complete BrainMap taxonomy) (Laird et al., 2009a). First, neuroimaging experiments in the BrainMap database that contained at least one activation focus within the ROI were extracted. Then, neuroimaging experiments were analyzed to determine the frequency of the behavioral domain of each ROI relative to the domain's likelihood across the entire BrainMap database (Eickhoff et al., 2011).

## Results

### Included studies and characteristics

A total of 118 experiments were incorporated into the present meta-analysis, comprising 96 fMRI experiments (from 92 studies) and 22 VBM experiments (from 19 studies). Among the fMRI experiments, 54 involved children (*N* = 977 for disorders, *N* = 949 for controls; mean age = 11 years) and 42 involved adults (*N* = 592 for disorders, *N* = 652 for controls; mean age = 27 years).

Among the VBM experiments, 16 involved children (*N* = 398 for disorders, *N* = 373 for controls; mean age = 11 years) and 6 involved adults (*N* = 81 for disorders, *N* = 82 for controls; mean age = 29 years). Detailed information for each study is provided in eTable 2 and eTable 3.

### Regional activation abnormalities across PDS and DD

**Fig. 2:**
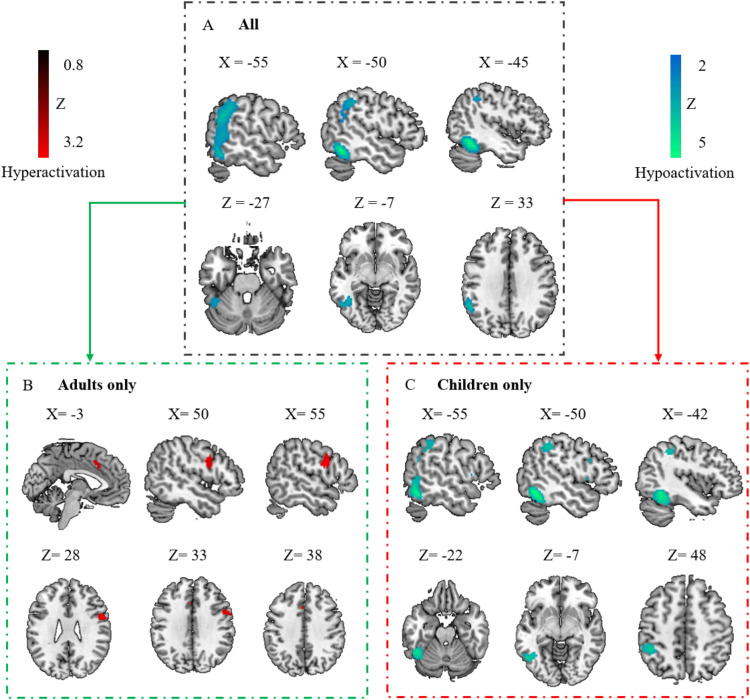
Regional activation abnormalities associated with the disorder group. Panel A shows regional activation abnormalities associated with individuals with DD and PDS. Panel B shows activation abnormalities associated with adults with disorders. Panel C shows activation abnormalities associated with children with disorders. Regions that survived with the statistical threshold set at *p*〈 0.005, a cluster extent of 10 voxels and the peak SDM-*Z* 〉 1. Coordinates reported in Montreal Neurological Institute space. **Abbreviation:** DD: developmental dyslexia; PDS: persistent developmental stuttering.

Taking PDS and DD as a whole, the results showed significant hypoactivation compared to controls in the left ITG, extending to the left SMG, IPG, and MTG (Fig. 2A and Table 1). No significant clusters of hyperactivation were found. Funnel plots (eFigure 1A) and Egger's test (*Z* = 0.57, *p* = 0.57) of the significant peak showed no significant publication bias. Low between-study heterogeneity was found for the significant peak (*I^2^* = 17.01 %).

**Table 1:** Coordinates of altered activation in the disorder group.

**Cluster**	**Voxels**	**Local peak**	**X**	**Y**	**Z**	**SDM-*Z***	**BA**
All DD and PDS							
*Hypoactivation*							
Left ITG	2085	Left ITG	−48	−56	−16	−5.17	37
		Left ITG	−46	−52	−18	−5.08	37
		Left fusiform gyrus	−42	−54	−14	−4.94	37
		Left SMG	−56	−50	30	−4.00	40
		Left SMG	−56	−52	26	−3.99	40
		Left IPG	−56	−48	38	−3.72	40
		Left IPG	−56	−44	38	−3.71	40
		Left MTG	−56	−54	0	−3.69	21
		Left MTG	−58	−46	6	−3.51	22
*Hyperactivation*							
No foci							
**Adults only**							
*Hypoactivation*							
No foci							
*Hyperactivation*							
Right PreCG	188	Right PreCG	54	2	26	3.26	6
		Right PreCG	54	6	28	3.15	6
		Right PreCG	54	6	34	3.02	6
Left MCC	43	Left MCC	−4	20	38	2.68	24
		Left MCC	−2	14	42	2.67	32
		Left SMA	−2	12	46	2.66	32
		Left MCC	−6	20	32	2.61	24
		Left MCC	−2	20	30	2.59	24
**Children only**							
*Hypoactivation*							
Left ITG	875	Left ITG	−50	−56	−16	−4.15	37
		Left ITG	−52	−58	−12	−4.04	37
		Left ITG	−46	−50	−24	−3.71	37
Left IPG	260	Left IPG	−46	−42	46	−3.45	40
		Left IPG	−56	−40	40	−2.94	40
		Left IPG	−56	−44	38	−2.91	40
Left PreCG	12	Left PreCG	−50	12	30	−2.68	44
Left MTG	11	Left MTG	−56	−54	22	−2.67	22
Left IFG, opercular part	10	Left IFG, opercular part	−52	10	8	−2.69	48
*Hyperactivation*							
No foci							

Regions that survived with the statistical threshold set at *p* 〈 0.005, a cluster extent of 10 voxels and the peak SDM-*Z* 〉 1. Coordinates reported in Montreal Neurological Institute space.

**Abbreviation:** BA: Broadman's area; IFG: inferior frontal gyrus; IPG: inferior parietal gyrus; ITG: inferior temporal gyrus; MCC: median cingulate cortex; MTG: middle temporal gyrus; PreCG: precentral gyrus; SMA: supplementary motor area; SMG: supramarginal gyrus.

When only participants with alphabetic languages were included, the main results were replicated (see eFigure 2 and eTable 4).

### Regional activation abnormalities in children and adults with DD and PDS

To address the potential impact of neurodevelopment, we conducted separate analyses for children and adults. Distinct patterns of regional activation deficits emerged across age groups. In adults, notable hyperactivation was identified in the right PreCG and left median cingulate cortex (MCC)/SMA (Fig. 2B and Table 1), whereas no significant clusters of hypoactivation were observed. Funnel plots (eFigure 1B&C) and Egger's test (*Z* = 0.41 and 0.26, *p* = 0.68 and 0.80 for the right PreCG and left MCC peak respectively) showed no significant publication bias. Low between-study heterogeneity was found for the significant peaks (*I^2^* = 4.97 % and 2.01 % for the right PreCG and left MCC, respectively).

In children with DD or PDS, significant hypoactivation manifested in the left ITG, IPG, PreCG, MTG, and IFG (Fig. 2C and Table 1), whereas no significant clusters of hyperactivation were identified. For three of these clusters (left ITG, MTG, and IFG), funnel plots (eFigure 1D&E&F) and Egger's test (*Z* = −1.88, −0.35, and −1.73, *p* = 0.06, 0.73 and 0.08 for the left ITG, MTG and IFG peak respectively) indicated no significant publication bias. For two of these clusters (the left IPG and PreCG), funnel plots (eFigure 1G&H) and Egger's test (*Z* = −2.05 and −3.73, *p* = 0.04 and *p* < 0.001 for the left IPG and PreCG peak respectively) showed significant publication bias, indicating the results may be driven by a small subset of studies or by studies with small sample sizes. Low to moderate between-study heterogeneity was found for the significant peaks (*I^2^* = 32.10 %, 0.46 %, 4.10 %, 7.12 %, and 26.29 % for the left ITG, MTG, IFG, IPG, and PreCG peak respectively).

### Alterations of gray matter across dd and PDS

No significant clusters were found when DD and PDS were analyzed together in the VBM analysis.

**Fig. 3:**
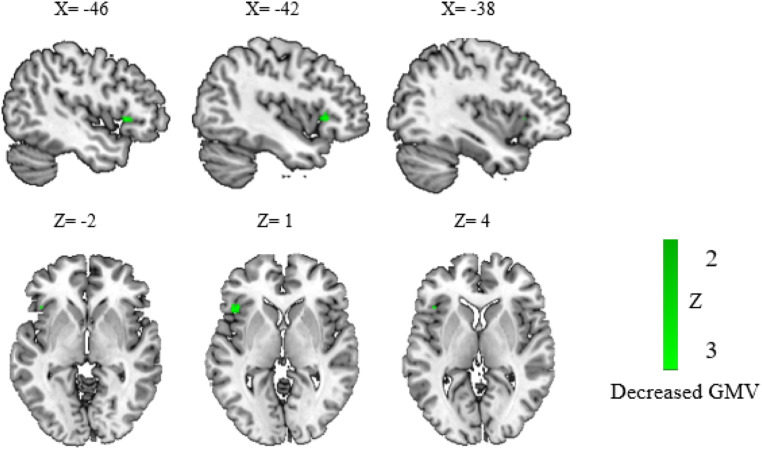
The results of structural atypical regions in children with disorders. Results showed a significant hypoactivation cluster of GMV in the left inferior frontal gyrus in children with DD and PDS compared to the control group. Regions that survived with the statistical threshold set at *p*〈 0.005, a cluster extent of 10 voxels and the peak SDM-*Z* 〉 1. Coordinates reported in Montreal Neurological Institute space. **Abbreviation:** DD: developmental dyslexia; GMV: gray matter volume; PDS: persistent developmental stuttering.

When child and adult participants were divided into subgroups, we identified a significant reduction of GMV in the left IFG (peak at −46, 20, 0; −42, 22, 0; −46, 44, −2) in children (Fig. 3). No significant clusters were found in adults. Funnel plots (eFigure 1I) and Egger's test (*Z* = −1.43, *p* = 0.15) showed no significant publication bias. Low between-study heterogeneity was found for the significant peak (*I^2^* = 9.87 %).

### MACM results

**Fig. 4:**
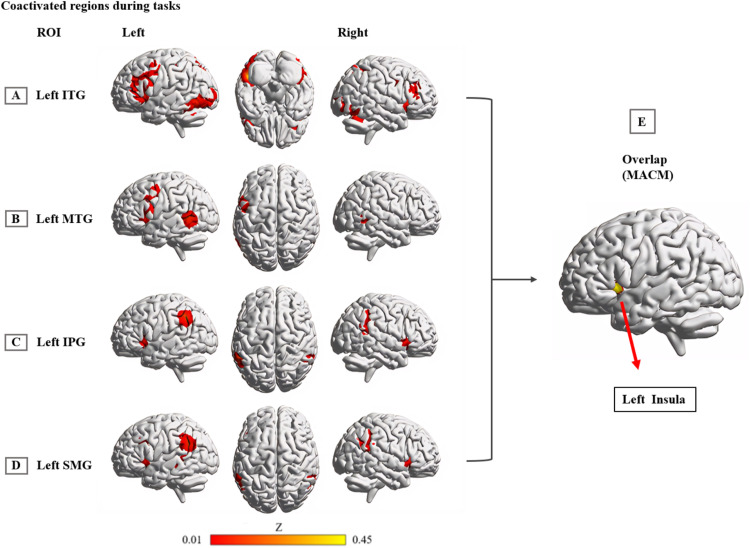
Interconnected brain network of common hypoactivation regions. Note: MACM results display brain regions that are coactivated with the left ITG (A), MTG (B), IPG (C) and SMG (D) in task-based activation studies of healthy participants from the BrainMap database, as well as a conjunction (E) across all 4 MACM maps. Coordinates reported in Montreal Neurological Institute space. **Abbreviation:** IPG: inferior parietal gyrus; ITG: inferior temporal gyrus; MACM: meta-analytic connectivity modeling; MTG: middle temporal gyrus; SMG: supramarginal gyrus.

The left ITG exhibited primary co-activation with bilateral frontal-parietal regions, including the bilateral MFG/IFG, insula, and inferior/superior parietal lobule (IPL/SPL). Additionally, co-activation extended to bilateral occipital-temporal regions, encompassing the bilateral fusiform gyrus, left middle occipital/temporal gyrus, and right inferior occipital gyrus (Fig. 4A. and eTable 5). The left MTG was predominately co-activated with the left MFG, IFG, insula, PreCG, ITG, and right MTG (Fig. 4B and eTable 6). The left IPG was mainly co-activated with the bilateral SMG, STG, and insula (Fig. 4C and eTable 7). The left SMG primarily co-activated with the left MTG, IFG, right IPL, bilateral insula, MFG, and right superior frontal gyrus (Fig. 4D and eTable 8).

Across all MACM results, the left insula region (MNI coordinates: −44, 16, 2; 125 voxels) exhibited consistent co-activation with the brain regions commonly associated with PDS and DD (Fig. 4E).

### Behavioral domain analysis results

The left ITG exhibited higher levels of activity during attention and semantic-related tasks (eFigure 3A). The left MTG showed enhanced activation, particularly during semantic-related tasks (eFigure 3B). The left IPG tended to display greater activity during attention-related tasks (eFigure 3C). The left SMG demonstrated increased activation during attention and social cognition-related tasks (eFigure 3D).

We also conducted a behavioral domain analysis on the common coactivation region of the left insula across the MACM results. The left insula tended to be more active during attention and speech tasks (eFigure 3E).

## Discussion

In this study, we employed a meta-analysis approach to identify shared brain signatures associated with PDS and DD, two prevalent developmental language disorders. First, by investigating task-related fMRI studies, we observed shared hypoactivation in the left ITG extending to the IPG when considering PDS and DD as a collective patient group, suggesting the shared function basis of spoken and written language impairments. Furthermore, when differentiating between children and adults, we found that the child group displayed significant hypoactivation in several language regions, including the left ITG, IPG, PreCG, MTG, and IFG, reflecting functional deficits in these developmental disorders. In contrast, the adult group exhibited hyperactivation in the motor cortex, suggesting a compensatory mechanism commonly related to language disorders. By investigating VBM studies, we found the anatomical alterations in the left IFG exclusively in children with DD or PDS, but not in adult participants, suggesting a shared structural basis for impairments in both written and spoken language disorders. Finally, we conducted a database-based analysis to explore the functional connectivity profiles of these shared brain regions and their related behaviors, aiming to understand how the functional and structural alterations contribute to language development disorders. This study, for the first time, revealed convergences in the functional and structural brain alterations associated with PDS and DD, providing new insights into the etiology of developmental language disorders.

### Shared functional hypoactivation across DD and PDS

We first searched shared functional signatures across language types by combining DD and PDS into a single patient group. We found significant hypoactivation in a large cluster centered at the left ITG extending to the MTG, suggesting a universal functional disruption across the two language disorders. The engagement of the ventral ITG in visual orthographic processing, including the VWFA, has been well-established in different written systems (Bolger et al., 2005; Martin et al., 2016; McCandliss et al., 2003; Price, 2012; Richlan et al., 2011; Van der Mark et al., 2009), although its specific role is debated (Price et al., 2011). Thus, it is understandable to observe the dysfunction of the VWFA in individuals with DD, as it is thought to underlie the impaired capacity for orthographic recognition (Siok et al., 2004). On the other hand, the involvement of the left ITG in both spoken and written language processing has been evidenced by lesion studies (Luders et al., 1991) and noninvasive neuroimaging studies (Demonet et al., 1992). Accordingly, the ITG has been considered a hub for integrating auditory and conceptual processing (Bonilha et al., 2017). Specifically, posterior lateral temporal regions were sensitive to early processing, linking auditory words to concepts, whereas anterior temporal regions were likely involved in additional and deeper levels of semantic processing (Bonilha et al., 2017). The aforementioned evidence is consistent with the ventral route of the sound-meaning mapping process (Hickok & Poeppel, 2007), which is indispensable to the perception and recognition of speech signals. Overall, the multifunctionality of the left ITG contributes to its crucial roles in accounting for both written and spoken language disorders. In this sense, our findings favor the hypothesis of VWFA as an interaction interface between visual orthographic information and high-level language (Price et al., 2011), rather than a pure region for housing visual word forms (Dehaene & Cohen, 2011).

Alternatively, beyond its role in language functions, the VWFA has recently been found to play a key role in attention. It is assumed to tune and amplify a range of visual stimuli, preparing them for use by other brain systems (Kay & Yeatman, 2017). Notably, reading and speaking tasks involve cognitive resources recruiting frontal and temporal regions (Peelle et al., 2004; Price, 2012). Thus, dysfunction of the VWFA may result in different manifestations of language disorders by affecting attention. Consistent with this perspective, we found coactivation between the left ITG and the left frontal-parietal attentional network in the MACM analysis.

In addition, we found diminished activation in dorsal parietal regions across DD and PDS, including IPG and SMG, suggesting that these regions may serve as a shared functional basis of different language disorders. Multiple fMRI studies have demonstrated the engagement of the left IPG in auditory-motor integration in speech perception or production, which is thought to facilitate the translation of speech representation from the STG to motor representations in the frontal lobe (Buchsbaum et al., 2001; Guenther, 2006; Hickok, 2012; Hickok et al., 2003; Wikman & Rinne, 2019). Additionally, the IPG has been reported to support the visual-motor integration process by coordinating visual information with motor execution processes (Batista et al., 1999; Kertzman et al., 1997; Ogawa & Inui, 2009; Sakata et al., 1995; Yuan & Brown, 2015). Collectively, these studies underscore the pivotal role of this parietal region in mediating sensory input and motor output, which may contribute to the normal development of language function. Thus, it is not surprising that individuals with language disorders exhibit dysfunction in the left IPG, as they often manifest sensory-motor integration and learning deficits. Specifically, individuals with PDS frequently show auditory-motor processing deficits (Assaneoet al., 2022; Cai et al., 2012; Terband et al., 2014; Watkins, 2011), while those with DD often show visual-motor processing deficits (Khatib et al., 2022; Qian & Bi, 2014),

Alternatively, like the VWFA, the IPG/SMG has been posited as a core region of the attention network (Corbetta & Shulman, 2002, 2011). Moreover, our behavioral-domain analysis indicated that this region is related to attention. Consequently, our findings illuminate the neural basis of the frequently observed attention problems in language disorders in behavioral studies (Crottaz-Herbette et al., 2004; Davachi et al., 2001; Noterdaeme & Amorosa, 1999; Petersen et al., 2013; Sciberras et al., 2014).

To further explore the functional significance of the shared regions and their relation to developmental language disorders (DD and PDS), we examined the functional connectivity patterns of these regions using MACM. In our analysis of ventral temporal and dorsal parietal ROIs, we observed consistent coactivation of the left insula. The insula has been conceptualized as a key hub connecting Broca's area, the superior temporal and inferior parietal cortex, linking the different codes of auditory perception (of the heard word), visual representation (of the written word), and articulatory sequence (of the spoken word) and converting between them (Paulesu et al., 1996). Functionally, lesion studies (Baratelli et al., 2015; Marxreiter et al., 2019; Peskine et al., 2008; Weiss et al., 2016) and fMRI studies (Ackermann & Riecker, 2004; Hickok & Poeppel, 2004; Riecker et al., 2000) have elucidated the roles of the insula in motor planning, speech motor control, auditory comprehension, and written language processes. Additionally, previous studies have demonstrated the role of the insula in domain-general cognitive functions, including executive control (Markostamou et al., 2015) and attention (Jarrahi et al., 2015; Kucyi et al., 2012; Qi et al., 2021; Wynn et al., 2015). Consistent with neuroimaging studies, behavioral-domain analysis also revealed predominant activation of the left insula during attention and language tasks. Together, the disruption of functional connectivity between the left insula and the left temporal-parietal region may represent universal critical neural circuits accounting for DD and PDS. Subsequent research should validate this proposed functional connectivity role in the two language disorders.

Furthermore, our findings have implications for the dual-route framework of speech, which postulates that the cortical architecture of speech perception bifurcates into the ventral stream and the dorsal stream (Hickok & Poeppel, 2004). The ventral stream traverses ventro-laterally towards the inferior posterior temporal cortex, supporting the mapping of sound onto meaning. The dorsal stream encompasses a region at the parietal-temporal juncture, eventually terminating in frontal regions, supporting the mapping of sound onto articulatory-based representations. The co-occurrence of dysfunction in both the ITG and IPG suggests that individuals with PDS and DD may face challenges in effectively integrating the ventral and dorsal routes, leading to compounded difficulties in reading, speaking, and overall language processing. Overall, these findings support the dual-route model by illuminating how disruptions in specific brain regions can affect both written and spoken language processing, emphasizing the need for targeted interventions that address both routes in individuals with language disorders.

### Shared functional hyperactivation patterns related to DD and PDS

In our secondary meta-analyses, we found common hyperactivation abnormalities in adult participants in the right PreCG, left SMA, and MCC/ACC. Overactivation in the right hemisphere is frequently attributed to increased effort and compensatory strategies, reflecting the development of complementary mechanisms to address deficits in the left language network (Bach et al., 2010; Martin et al., 2016; Richlan et al., 2009). This hyperactivation pattern aligns with the perspective that less efficient processing involves greater "tissue use" (Hare et al., 2008; Poldrack, 2015), suggesting a common compensatory mechanism in language impairment and related conditions (Cardebat et al., 1994; Cocquyt et al., 2017; Dick et al., 2013; Kraegeloh-Mann, 2004). Neuroimaging studies have provided evidence of the involvement of the PreCG in motor control, motor execution, and articulatory processes (Bach et al., 2010; Jackson et al., 2019; Petersen et al., 1988; van Ermingen-Marbach et al., 2013). Increased brain activation in the right PreCG in individuals with DD or PDS may indicate compensation for reliance on motor-articulation (Cao et al., 2017; Paulesu et al., 2014).

In addition, the MCC/ACC and SMA are motor cortices primarily involved in planning motor sequences (Shibasaki et al., 1993; Tanji, 2001; Tanji & Shima, 1994). Specifically, the cingulate gyrus is related to high-order motor control and monitors competition between responses, especially in task conditions eliciting conflict (Barch et al., 2000). The SMA plays a key role in the selection, planning, and production of voluntary hand movements, and it also contributes to speech perception, production, reading, and writing (Alario et al., 2006; Hertrich et al., 2016; Lima et al., 2016; Longcamp et al., 2019). Additionally, the left SMA is associated with verbal short-term memory and phonological rehearsal (Crottaz-Herbette et al., 2004; Davachi et al., 2001), suggesting that higher activation may assist individuals with DD or PDS in memorizing speeches and phonological structures. Finally, the SMA has been implicated in supporting sequential processing (Cona & Semenza, 2017), a critical component commonly involved in reading and speaking. Thus, the hyperactivation of this region may reflect deficits in sequential processing related to language development, in accordance with the procedural learning deficit hypothesis of developmental language disorders (Ullman et al., 2020).

A perceptual-motor theory of speech perception connects perceptual shaping and motor procedural knowledge in the human brain (Schwartz et al., 2012). Neurophysiological studies support the coupling of motor and sensory representations during speech perception and visual processing of letters (Fadiga et al., 2002; James & Gauthier, 2006; Nakatsuka et al., 2012). Thus, we propose that individuals with different language disorders, whether related to written or spoken language, may rely on motor strategies for compensating their language deficits during development.

Despite the finding that children with these two disorders exhibited reduced activation in several regions, we should be cautious about the result because no studies specifically involving children with PDS were included. The limited number of child studies leads us to speculate that the current findings are likely driven by the results of DD. Therefore, we did not discuss the results for children, and further studies are needed to obtain reliable insights into the dysfunction associated with language disorders in children.

### The shared anatomical basis of DD and PDS

The meta-analysis of VBM studies failed to reveal any shared anatomical alterations when combining both groups. However, in a secondary analysis separating children and adults, we found that the left IFG exhibited reduced GMV in children with written and spoken language disorders. A structural MRI analysis of an inherited speech and language disorder found reduced gray matter in the left IFG (Watkins et al., 2002), suggesting similar structural changes in individuals with spoken and written language disorders. Abundant evidence suggests that the left IFG is related to semantic, speech, phonological processing, and auditory-articulatory mapping (Beal et al., 2013; Booth et al., 2007; Tomaiuolo et al., 2021). A meta-analysis of healthy individuals found the left IFG (BA45, BA47) activation during both phonemic and semantic fluency tasks, regardless of whether the design was overt or covert (Wagner et al., 2014). Notably, all language disorders exhibit core dysfluency features during speaking for PDS (Yairi & Ambrose, 1999) or reading for DD (Lyon et al., 2003), potentially stemming from atypical structural problems in the left IFG.

However, we did not find overlaps between the functional and anatomical alterations in these two disorders. The relationship between structure and function is a fundamental question in neuroscience. The structural connectome shapes and constrains signaling transmission between neuronal populations, resulting in complex neuronal coactivation patterns that support perception, cognition, and other mental functions (Bullmore & Sporns, 2009; Yang et al., 2023). The prevailing hypothesis suggests that the structure-function relationship may gradually decouple from unimodal to transmodal cortex (Suárez et al., 2020), indicating that structure-function decoupling may not be an inherent characteristic of brain architecture (Yang et al., 2023). Given that language processing requires integration across modalities, such as sensory-motor modality combination and corresponding modality under different language modes (Li et al., 2022; Pattamadilok et al., 2019; Schaeffner et al., 2016), our finding of no overlap between structural and functional abnormalities is reasonable.

### Implications and future perspectives

This study identified common brain signatures for DD and PDS, implying the inherent connection between auditory and visual language modalities. Early developmental studies have demonstrated interactions between different language modalities, even if they exhibit uneven rates of development (Berninger, 2000; Berninger & Abbott, 2010). Behavioral studies show that phonological information efficiently enhances letter recognition performance and modulates the brain's response to visually presented stimuli (Arguin & Bub, 1995; Madec et al., 2016; Ziegler et al., 2000). Moreover, fMRI studies investigating neural substrates in different language modes have identified common activation in the premotor cortex during the processing of spoken and written languages (Longcamp et al., 2019). These findings collectively suggest the intertwined nature of different language modes, supporting our discovery of a common neural basis among DD and PDS.

In addition, the present results carry profound implications for further genetic research. Numerous genetic studies have identified a shared genetic architecture that significantly contributes to the susceptibility to both spoken and written language impairments, such as FOXP2, CNTNAP2, and CMIP (Graham & Fisher, 2013; Newbury et al., 2010; Paracchini, 2011; Whitehouse et al., 2011). Combining genetic analysis and neuroimaging, other studies have implicated language-related genes, such as Doublecortin Domain Containing 2 (DCDC2) and CNTNAP2, in gray matter distribution in language-related brain regions, including the ITG (Meda et al., 2008; Paternicó et al., 2016). Enlightened by these results, certain genes originating from specific core brain circuits are expected to serve as pivotal nodes within gene networks intricately linked to spoken and written language phenotypes.

### Limitations

The current meta-analysis has several limitations. First, the meta-analysis on children with DD or PDS found significant hypoactivation in the left IPG and PreCG, which was likely driven by a small subset of studies, as shown by asymmetry funnel plots (eFigure 1G&H). Therefore, it should be noted that this part of results should be interpreted with caution. Second, different linguistic tasks were used in the included studies, which might bias the results of our study. The participants with DD primarily engaged in reading-related tasks, while individuals with PDS typically focused on speech production activities (speaking), potentially eliciting distinct patterns of brain activation dependent on the specific task. However, it is noteworthy that despite the disparate tasks employed, our meta-analysis still identified common brain regions, highlighting the existence of fundamental language processing mechanisms underlying DD and PDS that transcend individual task demands. Furthermore, fMRI studies have illuminated a similar underlying neural network in both reading and speaking, encompassing a frontal-parietal-temporal language network, such as the inferior frontal gyrus (Sahin et al., 2009), and the left occipitotemporal areas (Longcamp et al., 2019; Qin et al., 2021), which to some extent reduce the impact of different tasks. Given that only a limited number of studies with similar tasks were included in the present meta-analysis, additional work is needed to examine the influence of different tasks on the results. Third, the results may be limited by the imbalance of studies between DD and PDS. To address this issue, the initial meta-analysis included the type of language disorders as a covariate for both fMRI and VBM data. Possibly, the VBM meta-analysis on adults, which encompassed only six experiments, may lack sufficient statistical power to detect decreased GMV. Future VBM studies on adults with DD or PDS are expected to provide further insights. Fourth, the results of the present analysis may be influenced by language bias, given that only English publications were included.

## Conclusions

The present study revealed shared brain abnormalities across DD and PDS, two representative disorders of written and spoken language, respectively. Functionally, we found that children and adults exhibited shared hypoactivation in the left ITG extending to the IPG. When differentiating between children and adults, children with DD or PDS exhibited shared dysfunction in language-related regions, while adults with DD or PDS exhibited compensatory hyperactivation in the motor cortex. Anatomically, we identified decreased GMV only in the left IFG in children with DD or PDS. Indeed, our findings do not imply that phenotypic differences between language disorders are negligible. Rather, identifying common neural markers sheds new light on the neurological models of the causes of developmental language disorders, and meanwhile emphasizes the importance of transdiagnostic neural signatures in language disorders.

## Declaration of competing interest

The authors declare that they have no known competing financial interests or personal relationships that could have appeared to influence the work reported in this paper.

## Appendix Supplementary materials

Image, application 1
