# Supplementary material for: The shared neurobiological basis of developmental dyslexia and developmental stuttering: A meta-analysis of functional and structural MRI studies
Source: Int J Clin Health Psychol. 2024 Nov 10;24(4):100519. doi: 10.1016/j.ijchp.2024.100519 (PMC11585698; doi:10.1016/j.ijchp.2024.100519)
Supplement: Supplementary file 1 [file mmc1.docx]

**The Shared Neurobiological Basis of Developmental Dyslexia and Developmental Stuttering: A Meta-Analysis of Functional and Structural MRI Studies**

**Supplemental Material**

**eTable 1**. PRISMA 2020 checklist

**eTable 2**. Information of selected functional magnetic resonance imaging studies

**eTable 3**. Information of selected voxel-based morphometry studies

**eTable 4**. Regional activation abnormalities associated with the disorder group under alphabetic languages

**eTable 5**. Meta-analytic connectivity modeling for the left inferior temporal gyrus

**eTable 6**. Meta-analytic connectivity modeling for the left middle temporal gyrus

**eTable 7**. Meta-analytic connectivity modeling for the left inferior parietal gyrus

**eTable 8**. Meta-analytic connectivity modeling for the left supramarginal gyrus

**eFigure 1**. Funnel plots

**eFigure 2**. Regional activation abnormalities associated with the disorder group under alphabetic languages

**eFigure 3**. Results of the behavioral domain meta-analysis

**eTable 1. PRISMA 2020 checklist**

| **Section and Topic** | **Item #** | **Checklist item** | **Location where item is reported** |
| --- | --- | --- | --- |
| **TITLE** | | |  |
| Title | 1 | Identify the report as a systematic review. | Pg 1 |
| **ABSTRACT** | | |  |
| Abstract | 2 | See the PRISMA 2020 for Abstracts checklist. | Pg 1 |
| **INTRODUCTION** | | |  |
| Rationale | 3 | Describe the rationale for the review in the context of existing knowledge. | Pg 1-2 |
| Objectives | 4 | Provide an explicit statement of the objective(s) or question(s) the review addresses. | Pg 2 |
| **METHODS** | | |  |
| Eligibility criteria | 5 | Specify the inclusion and exclusion criteria for the review and how studies were grouped for the syntheses. | Pg 2-3 |
| Information sources | 6 | Specify all databases, registers, websites, organisations, reference lists and other sources searched or consulted to identify studies. Specify the date when each source was last searched or consulted. | Pg 2-3 |
| Search strategy | 7 | Present the full search strategies for all databases, registers and websites, including any filters and limits used. | Pg 2-3 |
| Selection process | 8 | Specify the methods used to decide whether a study met the inclusion criteria of the review, including how many reviewers screened each record and each report retrieved, whether they worked independently, and if applicable, details of automation tools used in the process. | Pg 2-3 |
| Data collection process | 9 | Specify the methods used to collect data from reports, including how many reviewers collected data from each report, whether they worked independently, any processes for obtaining or confirming data from study investigators, and if applicable, details of automation tools used in the process. | Pg 2-3 |
| Data items | 10a | List and define all outcomes for which data were sought. Specify whether all results that were compatible with each outcome domain in each study were sought (e.g. for all measures, time points, analyses), and if not, the methods used to decide which results to collect. | Pg 2-3 |
|  | 10b | List and define all other variables for which data were sought (e.g. participant and intervention characteristics, funding sources). Describe any assumptions made about any missing or unclear information. | Pg 2-3 |
| Study risk of bias assessment | 11 | Specify the methods used to assess risk of bias in the included studies, including details of the tool(s) used, how many reviewers assessed each study and whether they worked independently, and if applicable, details of automation tools used in the process. | Pg 3-4 |
| Effect measures | 12 | Specify for each outcome the effect measure(s) (e.g. risk ratio, mean difference) used in the synthesis or presentation of results. | Pg 3-4 |
| Synthesis methods | 13a | Describe the processes used to decide which studies were eligible for each synthesis (e.g. tabulating the study intervention characteristics and comparing against the planned groups for each synthesis (item #5)). | Pg 3-4 |
|  | 13b | Describe any methods required to prepare the data for presentation or synthesis, such as handling of missing summary statistics, or data conversions. | Pg 3-4 |
|  | 13c | Describe any methods used to tabulate or visually display results of individual studies and syntheses. | Pg 3-4 |
|  | 13d | Describe any methods used to synthesize results and provide a rationale for the choice(s). If meta-analysis was performed, describe the model(s), method(s) to identify the presence and extent of statistical heterogeneity, and software package(s) used. | Pg 3-4 |
|  | 13e | Describe any methods used to explore possible causes of heterogeneity among study results (e.g. subgroup analysis, meta-regression). | Pg 3-4 |
|  | 13f | Describe any sensitivity analyses conducted to assess robustness of the synthesized results. | NA |
| Reporting bias assessment | 14 | Describe any methods used to assess risk of bias due to missing results in a synthesis (arising from reporting biases). | Pg 3-4 |
| Certainty assessment | 15 | Describe any methods used to assess certainty (or confidence) in the body of evidence for an outcome. | Pg 3-4 |
| **RESULTS** | | |  |
| Study selection | 16a | Describe the results of the search and selection process, from the number of records identified in the search to the number of studies included in the review, ideally using a flow diagram. | Pg 3; Fig. 1 |
|  | 16b | Cite studies that might appear to meet the inclusion criteria, but which were excluded, and explain why they were excluded. | NA |
| Study characteristics | 17 | Cite each included study and present its characteristics. | eTable 2&3 |
| Risk of bias in studies | 18 | Present assessments of risk of bias for each included study. | NA |
| Results of individual studies | 19 | For all outcomes, present, for each study: (a) summary statistics for each group (where appropriate) and (b) an effect estimate and its precision (e.g. confidence/credible interval), ideally using structured tables or plots. | Fig.2-4 Table1 |
| Results of syntheses | 20a | For each synthesis, briefly summarise the characteristics and risk of bias among contributing studies. | Pg 4-5 |
|  | 20b | Present results of all statistical syntheses conducted. If meta-analysis was done, present for each the summary estimate and its precision (e.g. confidence/credible interval) and measures of statistical heterogeneity. If comparing groups, describe the direction of the effect. | Pg 4-5  eFigure 1 |
|  | 20c | Present results of all investigations of possible causes of heterogeneity among study results. | Pg 4-5  eFigure 1 |
|  | 20d | Present results of all sensitivity analyses conducted to assess the robustness of the synthesized results. | NA |
| Reporting biases | 21 | Present assessments of risk of bias due to missing results (arising from reporting biases) for each synthesis assessed. | Pg 4-5 |
| Certainty of evidence | 22 | Present assessments of certainty (or confidence) in the body of evidence for each outcome assessed. | Pg 4-5 |
| **DISCUSSION** | | |  |
| Discussion | 23a | Provide a general interpretation of the results in the context of other evidence. | Pg 5-6 |
|  | 23b | Discuss any limitations of the evidence included in the review. | Pg 9 |
|  | 23c | Discuss any limitations of the review processes used. | Pg 9 |
|  | 23d | Discuss implications of the results for practice, policy, and future research. | Pg 9 |
| **OTHER INFORMATION** | | |  |
| Registration and protocol | 24a | Provide registration information for the review, including register name and registration number, or state that the review was not registered. | NA |
|  | 24b | Indicate where the review protocol can be accessed, or state that a protocol was not prepared. | NA |
|  | 24c | Describe and explain any amendments to information provided at registration or in the protocol. | NA |
| Support | 25 | Describe sources of financial or non-financial support for the review, and the role of the funders or sponsors in the review. | Funding |
| Competing interests | 26 | Declare any competing interests of review authors. | Declaration of Interest Statement |
| Availability of data, code and other materials | 27 | Report which of the following are publicly available and where they can be found: template data collection forms; data extracted from included studies; data used for all analyses; analytic code; any other materials used in the review. | Data sharing statement |

*From:*  Page MJ, McKenzie JE, Bossuyt PM, Boutron I, Hoffmann TC, Mulrow CD, et al. The PRISMA 2020 statement: an updated guideline for reporting systematic reviews. BMJ 2021;372:n71. doi: 10.1136/bmj.n71

**eTable 2. Information of selected functional magnetic resonance imaging studies**

| **Cit#** | **Study** | **Type of disorder** | **N_disorder** | **N_**  **TD** | **Age (mean)** | **Age**  **(SD)** | **Subject type** | **Task** | **Voxel level threshold** | | **Cluster level threshold** | **N_hypo** | **N_hyper** |
| --- | --- | --- | --- | --- | --- | --- | --- | --- | --- | --- | --- | --- | --- |
| 1 | Bach et al., (2010) | DD ^a^ | 14 | 18 | 8 | 0.35 | C | Covert reading task | *P<*0.005 | | 24 voxels | 2 | 0 |
| 2 | Banfi et al., (2020) | DD ^a^ | 20 | 22 | 9 | 4.4 | C | Covert reading task | FWE *P<*0.05 | | 10 voxels | 14 | 26 |
| 3 | Beneventi et al., (2009) | DD ^a^ | 11 | 13 | 13 | 0.5 | C | Letter probe task | *P<*0.001 | | 10 voxels | 3 | 0 |
| 4 | Beneventi et al., (2010) | DD ^a^ | 11 | 13 | 13 | 0.5 | C | 0-back working memory task | FDR *P<*0.05 | | 5 voxels | 4 | 0 |
| 5 | Booth et al., (2007) | DD ^a^ | 13 | 13 | 11 | 2.3 | C | Visual word judgment task | *P<*0.001 | | 15 voxels | 1 | 1 |
| 6 | Borghesani et al., (2021) | DD ^a^ | 26 | 14 | 10 | 1.8 | C | Word decision task | *P<*0.001 | | 100 voxels | 3 | 0 |
| 7 | Boros et al., (2016) | DD ^a^ | 15 | 18 | 12 | NR | C | Passive reading task | *P<*0.001 | | FDR *P<*0.05 | 6 | 0 |
| 8 | Brambati et al., (2006) | DD ^a^ | 13 | 11 | 31 | NR | A | Word reading and pseudoword reading tasks | *P<*0.005 | | 20 voxels | 9 | 0 |
| 9 | Brem et al., (2020) | DD ^a^ | 55 | 73 | 10 | 1.6 | C | Symbol string decision task | *P<*0.005 | | FWE *P<*0.05 | 0 | 2 |
| 10 | Cao et al., (2006) | DD ^a^ | 14 | 14 | 12 | NR | C | Visual word rhyming task | *P<*0.001 | | 15 voxels | 6 | 0 |
| 11 | Cao et al., (2008) | DD ^a^ | 12 | 12 | 12 | NR | C | Visual word rhyming task | *P<*0.001 | | 10 voxels | 5 | 1 |
| 12 | Cao et al., (2017) | DD ^m^ | 14 | 17 | 11 | NR | C | Auditory rhyming task | *P<*0.001 | | FDR *P<*0.05 | 5 | 3 |
| 13 | Cao et al., (2018) | DD ^m^ | 23 | 19 | 11 | NR | C | Visual spelling task | *P<*0.001 | | FDR *P<*0.05 | 6 | 5 |
| 14 | Cao et al., (2021) | DD ^m^ | 20 | 17 | 11 | NR | C | Visual rhyming task | *P<*0.001 | | FDR *P<*0.05 | 3 | 0 |
| 15 | Chang et al., (2009) | PDS ^a^ | 20 | 20 | 36 | 11 | A | Speech production task | | *P<*0.01 | NR | 10 | 15 |
| 16 | Christodoulou et al., (2014) | DD ^a^ | 12 | 12 | 23 | 3.6 | A | Sentence reading task | | *P<*0.001 | FDR *P<*0.01 | 4 | 0 |
| 17 | Chyl et al., (2019) | DD ^a^ | 25 | 25 | 9 | 0.5 | C | Visual word reading task | | *P<*0.001 | 50 voxels | 5 | 0 |
| 18 | Conant et al., (2020) | DD ^a^ | 12 | 13 | 13 | 2 | C | Phonemic test task | | *P<*0.01 | *P<*0.05 | 0 | 3 |
| *continued* | | | | | | | | | | | | | |
| 19 | Connally et al., (2018) | PDS ^a^ | 17 | 16 | 32 | 11.2 | A | Sentence reading and picture description tasks | | *P<*0.01 | 30 voxels | 16 | 8 |
| 20 | Conway et al., (2008) | DD ^a^ | 11 | 11 | 35 | 9.57 | A | Pseudowords-segmentation task | | *P<*0.005 | 150 ml | 0 | 1 |
| 21 | Cutting et al., (2013) | DD ^a^ | 20 | 19 | 12 | 0.3 | C | Lexical decision task | | *P<*0.005 | 34 voxels | 13 | 0 |
| 22 | Danelli et al., (2017) | DD ^a^ | 20 | 23 | 21 | 3.75 | A | Pseudoword reading task | | *P<*0.001 | FWE *P<*0.05 | 2 | 0 |
| 23 | Debska et al., (2021) | DD ^a^ | 38 | 42 | 10 | 0.94 | C | Word-symbol contrasts task | | *P<*0.005 | FWE *P<*0.05 | 2 | 0 |
| 24 | De Nil et al., (2008) | PDS ^a^ | 15 | 15 | 32 | 7.35 | A | Overt speech repetition task | | *P<*0.001 | NR | 3 | 1 |
| 25 | Desroches et al., (2010) | DD ^a^ | 12 | 12 | 11 | 2.25 | C | Auditory rhyming task | | *P<*0.001 | 15 voxels | 1 | 0 |
| 26 | Eden et al., (2004) | DD ^a^ | 19 | 19 | 43 | 9.55 | A | Word repetition task and initial sound deletion task | | *P<*0.001 | 80 voxels | 9 | 0 |
| 27 | Farris et al., (2016) | DD ^a^ | 16 | 15 | 9 | NR | C | Object rhyming task | | *P<*0.001 | 10 voxels | 2 | 1 |
| 28 | Feng et al., (2016) | DD ^m^ | 14 | 20 | 10 | 0.8 | C | Character spelling task | | *P<*0.001 | 39 voxels | 0 | 3 |
| 29 | Garnett et al., (2022) | PDS ^a^ | 15 | 16 | 22 | 6.45 | A | Solo reading task | | *P<*0.001 | *P<*0.05 | 6 | 4 |
| 30 | Georgiewa et al., (1999) | DD ^a^ | 17 | 17 | 14 | NR | C | Nonwords reading task | | *P<*0.05 | *P<*0.05 | 2 | 2 |
| 31 | Grande et al., (2011) | DD ^a^ | 25 | 20 | 10 | 0.75 | C | Words reading task | | *P<*0.001 | 10 voxels | 2 | 0 |
| 32 | Grunling et al., (2004) | DD ^a^ | 17 | 21 | 14 | 1.44 | C | Pseudoword rhyming task | | *P<*0.01 | 10 voxels | 3 | 31 |
| 33 | Hancock et al., (2016) | DD ^a^ | 16 | 11 | 10 | 1.2 | C | Word rhyming task | | *P<*0.01 | 50 voxels | 5 | 0 |
| 34 | Heim et al., (2010) | DD ^a^ | 20 | 20 | 9 | NR | C | First sound detection task | | *P<*0.001 | *P<*0.05 | 2 | 0 |
| 35 | Heim et al., (2013) | DD ^a^ | 11 | 15 | 36 | NR | A | Overt word reading task | | *P<*0.05 | NR | 1 | 0 |
| 36 | Heim et al., (2014) | DD ^a^ | 10 | 33 | 10 | 0.6 | C | Overt word reading task | | FWE *P<*0.05 | 100 voxels | 10 | 0 |
| 37 | Hernandez et al., (2013) | DD ^a^ | 15 | 16 | 21 | 2.15 | A | Word rhyming task | | *P<*0.001 | *P<*0.05 | 0 | 4 |
| 38 | Hoeft et al., (2006) | DD ^a^ | 10 | 10 | 10 | 1.3 | C | Visual word rhyming task | | *P<*0.001 | 10 voxels | 6 | 0 |
| 39 | Hoeft et al., (2007) | DD | 19 | 19 | 14 | 2.15 | C | Visual word rhyming task | | *P<*0.001 | NR | 4 | 7 |
| *continued* | | | | | | | | | | | | | |
| 40 | Howell et al., (2012) | PDS ^m^ | 9 | 9 | 24 | NR | A | Picture naming task | | *P<*0.005 | 311 voxels | 2 | 3 |
| 41 | Hu et al., (2010) | DD ^m^ | 8 | 8 | 14 | NR | C | Sematic word matching task | | *P<*0.001 | NR | 6 | 1 |
| 41 | Hu et al., (2010) | DD ^a^ | 11 | 10 | 14 | NR | C | Sematic word matching task | | *P<*0.001 | NR | 6 | 1 |
| 42 | Kast et al., (2011) | DD ^a^ | 12 | 13 | 26 | 4.65 | A | Lexical decision task | | *P<*0.001 | 30 voxels | 0 | 7 |
| 43 | Kell et al., (2009) | PDS ^a^ | 13 | 13 | 29 | NR | A | Overt reading task | | *P<*0.001 | *P<*0.05 | 4 | 14 |
| 44 | Kovelman et al., (2012) | DD ^a^ | 12 | 12 | 9 | 16.2 | C | Words rhyming task | | *P<*0.001 | 25 voxels | 1 | 0 |
| 45 | Kronbichler et al., (2006) | DD ^a^ | 13 | 15 | 16 | 0.51 | A | Sentence verification task | | FDR *P<*0.05 | 4 voxels | 2 | 13 |
| 46 | Kronschnabel et al., (2013) | DD ^a^ | 13 | 22 | 16 | 0.6 | A | Rapid serial visual stimulation detect task | | *P<*0.005 | 160 voxels | 5 | 2 |
| 47 | Kronschnabel et al., (2014) | DD ^a^ | 13 | 22 | 16 | 0.4 | A | Target detection task (Auditory-Visual combination) | | *P<*0.005 | 160 voxels | 0 | 15 |
| 48 | Langer et al., (2015) | DD ^a^ | 15 | 15 | 10 | 1.5 | C | Sentence reading task | | *P<*0.005 | 50 voxels | 16 | 3 |
| 49 | Liu et al., (2013) | DD ^m^ | 14 | 14 | 12 | 0.42 | C | Lexical match task | | *P<*0.001 | 10 voxels | 2 | 0 |
| 50 | Lobier et al., (2014) | DD ^a^ | 12 | 12 | 23 | 3.4 | A | visual categorization of character task | | *P<*0.001 | 20 voxels | 2 | 0 |
| 51 | Lu et al., (2009) | PDS ^m^ | 9 | 9 | 24 | NR | A | Picture naming task | | *P<*0.05 | NR | 2 | 9 |
| 52 | Lu et al., (2010) | PDS ^m^ | 12 | 12 | 24 | NR | A | Picture naming task | | *P<*0.05 | 220 mm^3 | 5 | 16 |
| 53 | Lu et al., (2016) | PDS ^m^ | 13 | 13 | 24 | 1.85 | A | Speech production task | | *P<*0.005 | 398 mm^3 | 0 | 1 |
| 54 | Maurer et al., (2010) | DD ^a^ | 19 | 13 | 11 | 0.4 | C | Word matching task | | *P<*0.01 | 30 voxels | 3 | 0 |
| 55 | Meyler et al., (2008) | DD ^a^ | 23 | 12 | 11 | 0.5 | C | Sentence comprehension task | | *P<*0.002 | 10 voxels | 6 | 2 |
| 56 | Neef et al., (2018) | PDS ^a^ | 31 | 34 | 37 | 12.5 | A | Speech production task | | *Z* >2.3 | *P<*0.05 | 0 | 3 |
| 57 | Neumann et al., (2003) | PDS ^a^ | 5 | 16 | 32 | NR | A | Overt reading task | | *P<*0.05 | NR | 3 | 13 |
| *Continued* | | | | | | | | | | | | | |
| 58 | Neumann et al., (2017) | PDS ^a^ | 13 | 13 | 27 | NR | A | Overt reading task | | *P<*0.001 | FWE *P<*0.05 | 5 | 6 |
| 59 | Norton et al., (2014) | DD ^a^ | 39 | 14 | 10 | 1 | C | Word rhyme task | | *P<*0.001 | NR | 9 | 0 |
| 60 | Olulade et al., (2012) | DD ^a^ | 6 | 9 | 21 | 1.71 | A | Word rhyme task | | *P<*0.005 | 10 voxels | 17 | 2 |
| 61 | Olulade et al., (2015) | DD ^a^ | 16 | 12 | 10 | 2.5 | C | Implicit word reading task | | *P<*0.001 | 20 voxels | 2 | 0 |
| 62 | OzernovPalchik et al., (2021) | DD ^a^ | 18 | 19 | 27 | 6.3 | A | Language comprehension task | | *P<*0.05 | NR | 8 | 0 |
| 63 | Pekkola et al., (2006) | DD ^a^ | 10 | 10 | 28 | NR | A | Audio-visual speech perception task | | Z>1.8 | *P<*0.05 corrected | 0 | 4 |
| 64 | Perrachione et al., (2016) | DD ^a^ | 19 | 19 | 23 | 4.8 | A | Voice adaptation task | | *P<*0.001 | FDR *P<*0.001 | 2 | 0 |
| 64 | Perrachione et al., (2016) | DD ^a^ | 23 | 24 | 22 | 3.9 | A | Speech adaptation task | | *P<*0.001 | FDR *P<*0.001 | 2 | 0 |
| 64 | Perrachione et al., (2016) | DD ^a^ | 26 | 25 | 8 | 0.6 | C | Speech adaptation task | | *P<*0.001 | FDR *P<*0.05 | 1 | 0 |
| 65 | Prasad et al., (2020) | DD ^a^ | 16 | 15 | 12 | NR | C | Simple sentence reading task | | *P<*0.001 | 20 voxels | 2 | 3 |
| 66 | Preibisch et al., (2003) | PDS ^a^ | 16 | 16 | 31 | 9 | A | Sentence reading task | | *P<*0.001 | NR | 2 | 13 |
| 67 | Richlan et al., (2010) | DD ^a^ | 15 | 18 | 18 | 1.13 | A | Phonological lexical decision task | | *P<*0.005 | 38 voxels | 3 | 6 |
| 68 | Ruff et al., (2002) | DD ^a^ | 6 | 11 | 29 | 8 | A | Passive listening speech task | | *P<*0.01 | 38 voxels | 4 | 2 |
| 69 | Sakai et al., (2009) | PDS ^m^ | 8 | 10 | 27 | NR | A | Overt reading task | | *P<*0.01 | NR | 9 | 0 |
| 70 | Sares et al., (2020) | PDS ^a^ | 13 | 15 | 29 | 11.19 | A | Reading-vowel task | | *P<*0.001 | NR | 3 | 0 |
| 71 | Schulz et al., (2008) | DD ^a^ | 16 | 31 | 11 | 0.4 | C | Sentence reading task | | *P<*0.001 | NR | 6 | 0 |
| 72 | Schulz et al., (2009) | DD ^a^ | 19 | 19 | 12 | 0.35 | C | Sentence reading task | | *P<*0.001 | NR | 9 | 0 |
| 73 | Siok et al., (2004) | DD ^m^ | 8 | 8 | 11 | NR | C | Homophone judgment task | | *P<*0.001 | 20 voxels | 5 | 1 |
| 74 | Siok et al., (2008) | DD ^m^ | 12 | 12 | 11 | NR | C | Character rhyming task | | *P<*0.005 | 10 voxels | 14 | 0 |
| *continued* | | | | | | | | | | | | | |
| 75 | Siok et al., (2009) | DD ^m^ | 12 | 12 | 11 | NR | C | Font size judgment task | | FDR *P<*0.05 | 10 voxels | 2 | 1 |
| 76 | Tanaka et al., (2011) | DD ^a^ | 16 | 18 | 10 | 1 | C | Word rhyme task | | *P<*0.05 | NR | 2 | 0 |
| 77 | Temple et al., (2001) | DD ^a^ | 24 | 15 | 11 | 1.4 | C | Letter matching task | | *P<*0.001 | 20 voxels | 5 | 1 |
| 78 | Toyomura et al., (2011) | PDS ^m^ | 12 | 12 | 28 | 8.1 | A | Reading-sentence task | | *P<*0.001 | NR | 0 | 3 |
| 79 | Toyomura et al., (2015) | PDS ^m^ | 10 | 10 | 25 | 3.8 | A | Reading-sentence task | | FWE *P<*0.05 | 10 voxels | 7 | 0 |
| 80 | Toyomura et al., (2018) | PDS ^m^ | 18 | 18 | 29 | 9.1 | A | Speech production task | | *P<*0.001 | 10 voxels | 0 | 2 |
| 81 | vanderMark et al., (2009) | DD ^a^ | 24 | 18 | 11 | 0.6 | C | Phonological lexical decision task | | *P<*0.001 | 10 voxels | 8 | 0 |
| 82 | vanermingenmarbach et al., (2013) | DD ^a^ | 17 | 13 | 10 | NR | C | Phoneme detection task | | *P<*0.001 | 10 voxels | 0 | 7 |
| 82 | vanermingenmarbach et al., (2013) | DD ^a^ | 14 | 13 | 10 | NR | C | Phoneme detection task | | *P<*0.001 | 10 voxels | 0 | 7 |
| 83 | vanermingenmarbach et al., (2013) | DD ^a^ | 32 | 10 | 10 | 0.5 | C | Initial phoneme detection task | | *P<*0.01 | 30 voxels | 2 | 2 |
| 84 | waldie et al., (2013) | DD ^a^ | 12 | 16 | 30 | 8.3 | A | Regular word lexical decision task | | *P<*0.001 | NR | 1 | 14 |
| 85 | Wangfang et al., (2020) | DD ^a^ | 17 | 15 | 7 | 0.46 | C | Implicit target detection task | | *P<*0.005 | 50 voxels | 1 | 0 |
| 86 | Ward et al., (2015) | PDS ^a^ | 17 | 17 | 33 | NR | A | Reading-sentence task | | *P<*0.01 | 30 voxels | 1 | 14 |
| 87 | Watkins et al., (2008) | PDS ^a^ | 12 | 10 | 18 | NR | A | Speech production task | | *P<*0.001 | *P<*0.05 | 5 | 8 |
| 88 | Weiss et al., (2016) | DD ^a^ | 21 | 22 | 27 | 2.5 | A | Word reading task | | *P<*0.001 | 50 voxels | 1 | 0 |
| 89 | Wimmer et al., (2010) | DD ^a^ | 20 | 19 | 21 | 6.8 | A | Phonological lexical decision task | | *P<*0.005 | 10 voxels | 1 | 8 |
| 90 | Yang and Tan, (2020) | DD ^m^ | 16 | 16 | 10 | NR | C | Component judgments task | | *P<*0.005 | 25 voxels | 1 | 0 |
| 91 | Yang et al., (2022) | DD ^m^ | 18 | 23 | 10 | 0.55 | C | Character direct copying task | | *P<*0.001 | FWE *P<*0.05 | 17 | 5 |
| 92 | Yangyang et al., (2020) | DD ^m^ | 14 | 16 | 11 | 1 | C | Lexical decision task | | *P<*0.005 | 110 voxels | 4 | 0 |

Notes:

^a^: alphabetic language disorder; ^m^: morpho-syllabic language disorder.

**Abbreviation:**

A: adult; C: children; DD: developmental dyslexia; FDR: false discovery rate; FWE: family wise error; PDS: persistent developmental stuttering; NR: not reported; N_disorder: number of subjects with DD or PDS; N_TD: number of typical developing subjects; N_hypo: number of hypoactivated foci; N_hyper: number of hyperactivated foci.

**1.** Bach S, Brandeis D, Hofstetter C, Martin E, Richardson U, Brem S. Early emergence of deviant frontal fMRI activity for phonological processes in poor beginning readers. *Neuroimage.* Nov 1 2010;53(2):682-693.

**2.** Banfi C, Koschutnig K, Moll K, Schulte-Koerne G, Fink A, Landerl K. Reading-related functional activity in children with isolated spelling deficits and dyslexia. *LANGUAGE COGNITION AND NEUROSCIENCE.* 2021 JUN 1 2021;36(5):543-561.

**3.** Beneventi H, Tonnessen FE, Ersland L. DYSLEXIC CHILDREN SHOW SHORT-TERM MEMORY DEFICITS IN PHONOLOGICAL STORAGE AND SERIAL REHEARSAL: AN fMRI STUDY. *INTERNATIONAL JOURNAL OF NEUROSCIENCE.* 2009 2009;119(11):2017-2043.

**4.** Beneventi H, Tonnessen FE, Ersland L, Hugdahl K. Executive working memory processes in dyslexia: Behavioral and fMRI evidence. *SCANDINAVIAN JOURNAL OF PSYCHOLOGY.* 2010 JUN 2010;51(3):192-202.

**5.** Booth JR, Bebko G, Burman DD, Bitan T. Children with reading disorder show modality independent brain abnormalities during semantic tasks. *Neuropsychologia.* Mar 2 2007;45(4):775-783.

**6.** Borghesani V, Wang C, Watson C, et al. Functional and morphological correlates of developmental dyslexia: A multimodal investigation of the ventral occipitotemporal cortex. *J Neuroimaging.* Sep 2021;31(5):962-972.

**7.** Boros M, Anton J-L, Pech-Georgel C, Grainger J, Szwed M, Ziegler JC. Orthographic processing deficits in developmental dyslexia: Beyond the ventral visual stream. *NEUROIMAGE.* 2016 MAR 2016;128:316-327.

**8.** Brambati SM, Termine C, Ruffino M, et al. Neuropsychological deficits and neural dysfunction in familial dyslexia. *Brain Res.* Oct 3 2006;1113(1):174-185.

**9.** Brem S, Maurer U, Kronbichler M, et al. Visual word form processing deficits driven by severity of reading impairments in children with developmental dyslexia. *SCIENTIFIC REPORTS.* 2020 OCT 30 2020;10(1).

**10.** Cao F, Bitan T, Chou T-L, Burman DD, Booth JR. Deficient orthographic and phonological representations in children with dyslexia revealed by brain activation patterns. *JOURNAL OF CHILD PSYCHOLOGY AND PSYCHIATRY.* 2006 OCT 2006;47(10):1041-1050.

**11.** Cao F, Bitan T, Booth JR. Effective brain connectivity in children with reading difficulties during phonological processing. *Brain Lang.* Nov 2008;107(2):91-101.

**12.** Cao F, Yan X, Wang Z, et al. Neural signatures of phonological deficits in Chinese developmental dyslexia. *Neuroimage.* Feb 1 2017;146:301-311.

**13.** Cao F, Yan X, Spray GJ, Liu Y, Deng Y. Brain Mechanisms Underlying Visuo-Orthographic Deficits in Children With Developmental Dyslexia. *Front Hum Neurosci.* 2018;12:490.

**14.** Cao F, Yan XH, Yan X, Zhou HY, Booth JR. Reading Disability in Chinese Children Learning English as an L2. *CHILD DEVELOPMENT.* MAR 2021;92(2):E126-E142.

**15.** Chang S-E, Kenney MK, Loucks TMJ, Ludlow CL. Brain activation abnormalities during speech and non-speech in stuttering speakers. *NEUROIMAGE.* 2009 MAY 15 2009;46(1):201-212.

**16.** Christodoulou JA, Del Tufo SN, Lymberis J, et al. Brain Bases of Reading Fluency in Typical Reading and Impaired Fluency in Dyslexia. *PLOS ONE.* 2014 JUL 24 2014;9(7).

**17.** Chyl K, Kossowski B, Dębska A, et al. Reading Acquisition in Children: Developmental Processes and Dyslexia-Specific Effects. *J Am Acad Child Adolesc Psychiatry.* Oct 2019;58(10):948-960.

**18.** Conant LL, Liebenthal E, Desai A, Seidenberg MS, Binder JR. Differential activation of the visual word form area during auditory phoneme perception in youth with dyslexia. *Neuropsychologia.* Sep 2020;146:107543.

**19.** Connally EL, Ward D, Pliatsikas C, et al. Separation of trait and state in stuttering. *Hum Brain Mapp.* Aug 2018;39(8):3109-3126.

**20.** Conway T, Heilman KM, Gopinath K, et al. Neural substrates related to auditory working memory comparisons in dyslexia: an fMRI study. *J Int Neuropsychol Soc.* Jul 2008;14(4):629-639.

**21.** Cutting LE, Clements-Stephens A, Pugh KR, et al. Not All Reading Disabilities Are Dyslexia: Distinct Neurobiology of Specific Comprehension Deficits. *BRAIN CONNECTIVITY.* 2013 APR 2013;3(2):199-211.

**22.** Danelli L, Berlingeri M, Bottini G, et al. How many deficits in the same dyslexic brains? A behavioural and fMRI assessment of comorbidity in adult dyslexics. *Cortex.* Dec 2017;97:125-142.

**23.** Dębska A, Banfi C, Chyl K, et al. Neural patterns of word processing differ in children with dyslexia and isolated spelling deficit. *Brain Struct Funct.* Jun 2021;226(5):1467-1478.

**24.** De Nil LF, Beal DS, Lafaille SJ, Kroll RM, Crawley AP, Gracco VL. The effects of simulated stuttering and prolonged speech on the neural activation patterns of stuttering and nonstuttering adults. *Brain Lang.* Nov 2008;107(2):114-123.

**25.** Desroches AS, Cone NE, Bolger DJ, Bitan T, Burman DD, Booth JR. Children with reading difficulties show differences in brain regions associated with orthographic processing during spoken language processing. *Brain Res.* Oct 14 2010;1356:73-84.

**26.** Eden GF, Jones KM, Cappell K, et al. Neural changes following remediation in adult developmental dyslexia. *NEURON.* OCT 28 2004;44(3):411-422.

**27.** Farris EA, Ring J, Black J, Lyon GR, Odegard TN. Predicting Growth in Word Level Reading Skills in Children With Developmental Dyslexia Using an Object Rhyming Functional Neuroimaging Task. *Dev Neuropsychol.* Apr 2016;41(3):145-161.

**28.** Feng X, Li L, Zhang M, et al. Dyslexic Children Show Atypical Cerebellar Activation and Cerebro-Cerebellar Functional Connectivity in Orthographic and Phonological Processing. *Cerebellum.* Apr 2017;16(2):496-507.

**29.** Garnett EO, Chow HM, Limb S, Liu Y, Chang SE. Neural activity during solo and choral reading: A functional magnetic resonance imaging study of overt continuous speech production in adults who stutter. *Front Hum Neurosci.* 2022;16:894676.

**30.** Georgiewa P, Rzanny R, Hopf JM, et al. fMRI during word processing in dyslexic and normal reading children. *NEUROREPORT.* 1999 NOV 8 1999;10(16):3459-3465.

**31.** Grande M, Meffert E, Huber W, Amunts K, Heim S. Word frequency effects in the left IFG in dyslexic and normally reading children during picture naming and reading. *Neuroimage.* Aug 1 2011;57(3):1212-1220.

**32.** Gruenling C, Ligges M, Huonker R, et al. Dyslexia: the possible benefit of multimodal integration of fMRI- and EEG-data. *Journal of Neural Transmission.* 2004 2004;111(7):951-969.

**33.** Hancock R, Gabrieli JDE, Hoeft F. Shared temporoparietal dysfunction in dyslexia and typical readers with discrepantly high IQ. *Trends Neurosci Educ.* Dec 2016;5(4):173-177.

**34.** Heim S, Grande M, Pape-Neumann J, et al. Interaction of Phonological Awareness and 'Magnocellular' Processing During Normal and Dyslexic Reading: Behavioural and fMRI Investigations. *DYSLEXIA.* 2010 AUG 2010;16(3):258-282.

**35.** Heim S, Wehnelt A, Grande M, Huber W, Amunts K. Effects of lexicality and word frequency on brain activation in dyslexic readers. *BRAIN AND LANGUAGE.* 2013 MAY 2013;125(2):194-202.

**36.** Heim S, Weidner R, von Overheidt A-C, Tholen N, Grande M, Amunts K. Experimental induction of reading difficulties in normal readers provides novel insights into the neurofunctional mechanisms of visual word recognition. *BRAIN STRUCTURE & FUNCTION.* 2014 MAR 2014;219(2):461-471.

**37.** Hernandez N, Andersson F, Edjlali M, et al. Cerebral functional asymmetry and phonological performance in dyslexic adults. *PSYCHOPHYSIOLOGY.* 2013 DEC 2013;50(12):1226-1238.

**38.** Hoeft F, Hernandez A, McMillon G, et al. Neural basis of dyslexia: a comparison between dyslexic and nondyslexic children equated for reading ability. *J Neurosci.* Oct 18 2006;26(42):10700-10708.

**39.** Hoeft F, Meyler A, Hernandez A, et al. Functional and morphometric brain dissociation between dyslexia and reading ability. *Proc Natl Acad Sci U S A.* Mar 6 2007;104(10):4234-4239.

**40.** Howell P, Jiang J, Peng DL, Lu CM. Neural control of rising and falling tones in Mandarin speakers who stutter. *BRAIN AND LANGUAGE.* DEC 2012;123(3):211-221.

**41.** Hu W, Lee HL, Zhang Q, et al. Developmental dyslexia in Chinese and English populations: dissociating the effect of dyslexia from language differences. *Brain.* Jun 2010;133(Pt 6):1694-1706.

**42.** Kast M, Bezzola L, Jäncke L, Meyer M. Multi- and unisensory decoding of words and nonwords result in differential brain responses in dyslexic and nondyslexic adults. *Brain Lang.* Dec 2011;119(3):136-148.

**43.** Kell CA, Neumann K, von Kriegstein K, et al. How the brain repairs stuttering. *BRAIN.* 2009 OCT 2009;132:2747-2760.

**44.** Kovelman I, Norton ES, Christodoulou JA, et al. Brain basis of phonological awareness for spoken language in children and its disruption in dyslexia. *Cereb Cortex.* Apr 2012;22(4):754-764.

**45.** Kronbichler M, Hutzler F, Staffen W, Mair A, Ladurner G, Wimmer H. Evidence for a dysfunction of left posterior reading areas in German dyslexic readers. *Neuropsychologia.* 2006;44(10):1822-1832.

**46.** Kronschnabel J, Schmid R, Maurer U, Brandeis D. Visual print tuning deficits in dyslexic adolescents under minimized phonological demands. *Neuroimage.* Jul 1 2013;74:58-69.

**47.** Kronschnabel J, Brem S, Maurer U, Brandeis D. The level of audiovisual print-speech integration deficits in dyslexia. *NEUROPSYCHOLOGIA.* 2014 SEP 2014;62:245-261.

**48.** Langer N, Benjamin C, Minas J, Gaab N. The Neural Correlates of Reading Fluency Deficits in Children. *CEREBRAL CORTEX.* 2015 JUN 2015;25(6):1441-1453.

**49.** Liu L, Tao R, Wang W, You W, Peng D, Booth JR. Chinese dyslexics show neural differences in morphological processing. *Dev Cogn Neurosci.* Oct 2013;6:40-50.

**50.** Lobier MA, Peyrin C, Pichat C, Le Bas JF, Valdois S. Visual processing of multiple elements in the dyslexic brain: evidence for a superior parietal dysfunction. *Front Hum Neurosci.* 2014;8:479.

**51.** Lu C, Ning N, Peng D, et al. THE ROLE OF LARGE-SCALE NEURAL INTERACTIONS FOR DEVELOPMENTAL STUTTERING. *NEUROSCIENCE.* JUL 21 2009;161(4):1008-1026.

**52.** Lu C, Chen C, Ning N, et al. The neural substrates for atypical planning and execution of word production in stuttering. *EXPERIMENTAL NEUROLOGY.* 2010 JAN 2010;221(1):146-156.

**53.** Lu C, Long Y, Zheng L, et al. Relationship between Speech Production and Perception in People Who Stutter. *Front Hum Neurosci.* 2016;10:224.

**54.** Maurer U, Schulz E, Brem S, et al. The development of print tuning in children with dyslexia: evidence from longitudinal ERP data supported by fMRI. *Neuroimage.* Aug 1 2011;57(3):714-722.

**55.** Meyler A, Keller TA, Cherkassky VL, Gabrieli JDE, Just MA. Modifying the brain activation of poor readers during sentence comprehension with extended remedial instruction: A longitudinal study of neuroplasticity. *NEUROPSYCHOLOGIA.* 2008 AUG 2008;46(10):2580-2592.

**56.** Neef NE, Anwander A, Bütfering C, et al. Structural connectivity of right frontal hyperactive areas scales with stuttering severity. *Brain.* Jan 1 2018;141(1):191-204.

**57.** Neumann K, Euler HA, Kob M, et al. Assisted and unassisted recession of functional anomalies associated with dysprosody in adults who stutter. *JOURNAL OF FLUENCY DISORDERS.* 2018 MAR 2018;55:120-134.

**58.** Neumann K, Euler HA, von Gudenberg AW, et al. The nature and treatment of stuttering as revealed by fMRI A within- and between-group comparison. *J Fluency Disord.* Winter 2003;28(4):381-409; quiz 409-410.

**59.** Norton ES, Black JM, Stanley LM, et al. Functional neuroanatomical evidence for the double-deficit hypothesis of developmental dyslexia. *NEUROPSYCHOLOGIA.* 2014 AUG 2014;61:235-246.

**60.** Olulade OA, Gilger JW, Talavage TM, Hynd GW, McAteer CI. Beyond phonological processing deficits in adult dyslexics: atypical FMRI activation patterns for spatial problem solving. *Dev Neuropsychol.* 2012;37(7):617-635.

**61.** Olulade OA, Flowers DL, Napoliello EM, Eden GF. Dyslexic children lack word selectivity gradients in occipito-temporal and inferior frontal cortex. *NEUROIMAGE-CLINICAL.* 2015 2015;7:742-754.

**62.** Ozernov-Palchik O, Centanni TM, Beach SD, May S, Hogan T, Gabrieli JDE. Distinct neural substrates of individual differences in components of reading comprehension in adults with or without dyslexia. *NEUROIMAGE.* 2021 FEB 1 2021;226.

**63.** Pekkola J, Laasonen M, Ojanen V, et al. Perception of matching and conflicting audiovisual speech in dyslexic and fluent readers: an fMRI study at 3 T. *Neuroimage.* Feb 1 2006;29(3):797-807.

**64.** Perrachione TK, Del Tufo SN, Winter R, et al. Dysfunction of Rapid Neural Adaptation in Dyslexia. *Neuron.* Dec 21 2016;92(6):1383-1397.

**65.** Prasad S, Sagar R, Kumaran SS, Mehta M. Study of functional magnetic resonance imaging (fMRI) in children and adolescents with specific learning disorder (dyslexia). *Asian J Psychiatr.* Apr 2020;50:101945.

**66.** Preibisch C, Neumann K, Raab P, et al. Evidence for compensation for stuttering by the right frontal operculum. *Neuroimage.* Oct 2003;20(2):1356-1364.

**67.** Richlan F, Sturm D, Schurz M, Kronbichler M, Ladurner G, Wimmer H. A common left occipito-temporal dysfunction in developmental dyslexia and acquired letter-by-letter reading? *PLoS One.* Aug 11 2010;5(8):e12073.

**68.** Ruff S, Cardebat D, Marie N, Demonet JF. Enhanced response of the left frontal cortex to slowed down speech in dyslexia: an fMRI study. *NEUROREPORT.* 2002 JUL 19 2002;13(10):1285-1289.

**69.** Sakai N, Masuda S, Shimotomai T, Mori K. Brain activation in adults who stutter under delayed auditory feedback: An fMRI study. *INTERNATIONAL JOURNAL OF SPEECH-LANGUAGE PATHOLOGY.* 2009 FEB 2009;11(1):2-11.

**70.** Sares AG, Deroche MLD, Ohashi H, Shiller DM, Gracco VL. Neural Correlates of Vocal Pitch Compensation in Individuals Who Stutter. *Front Hum Neurosci.* 2020;14:18.

**71.** Schulz E, Maurer U, van der Mark S, et al. Impaired semantic processing during sentence reading in children with dyslexia: combined fMRI and ERP evidence. *Neuroimage.* May 15 2008;41(1):153-168.

**72.** Schulz E, Maurer U, van der Mark S, et al. Reading for meaning in dyslexic and young children: Distinct neural pathways but common endpoints. *NEUROPSYCHOLOGIA.* 2009 OCT 2009;47(12):2544-2557.

**73.** Siok WT, Perfetti CA, Jin Z, Tan LH. Biological abnormality of impaired reading is constrained by culture. *Nature.* Sep 2 2004;431(7004):71-76.

**74.** Siok WT, Niu Z, Jin Z, Perfetti CA, Tan LH. A structural-functional basis for dyslexia in the cortex of Chinese readers. *Proc Natl Acad Sci U S A.* Apr 8 2008;105(14):5561-5566.

**75.** Siok WT, Spinks JA, Jin Z, Tan LH. Developmental dyslexia is characterized by the co-existence of visuospatial and phonological disorders in Chinese children. *CURRENT BIOLOGY.* 2009 OCT 13 2009;19(19):R890-R892.

**76.** Tanaka H, Black JM, Hulme C, et al. The brain basis of the phonological deficit in dyslexia is independent of IQ. *Psychol Sci.* Nov 2011;22(11):1442-1451.

**77.** Temple E, Poldrack RA, Salidis J, et al. Disrupted neural responses to phonological and orthographic processing in dyslexic children: an fMRI study. *NEUROREPORT.* 2001 FEB 12 2001;12(2):299-307.

**78.** Toyomura A, Fujii T, Kuriki S. Effect of external auditory pacing on the neural activity of stuttering speakers. *Neuroimage.* Aug 15 2011;57(4):1507-1516.

**79.** Toyomura A, Fujii T, Kuriki S. Effect of an 8-week practice of externally triggered speech on basal ganglia activity of stuttering and fluent speakers. *Neuroimage.* Apr 1 2015;109:458-468.

**80.** Toyomura A, Fujii T, Yokosawa K, Kuriki S. Speech Disfluency-dependent Amygdala Activity in Adults Who Stutter: Neuroimaging of Interpersonal Communication in MRI Scanner Environment. *Neuroscience.* Mar 15 2018;374:144-154.

**81.** Van der Mark S, Bucher K, Maurer U, et al. Children with dyslexia lack multiple specializations along the visual word-form (VWF) system. *NEUROIMAGE.* 2009 OCT 1 2009;47(4):1940-1949.

**82.** van Ermingen-Marbach M, Grande M, Pape-Neumann J, Sass K, Heim S. Distinct neural signatures of cognitive subtypes of dyslexia with and without phonological deficits. *Neuroimage Clin.* 2013;2:477-490.

**83.** van Ermingen-Marbach M, Pape-Neumann J, Grande M, Grabowska A, Heim S. Distinct neural signatures of cognitive subtypes of dyslexia: Effects of lexicality during phonological processing. *ACTA NEUROBIOLOGIAE EXPERIMENTALIS.* 2013;73(3):404-416.

**84.** Waldie KE, Haigh CE, Badzakova-Trajkov G, Buckley J, Kirk IJ. Reading the wrong way with the right hemisphere. *Brain Sci.* Jul 17 2013;3(3):1060-1075.

**85.** Wang F, Karipidis, II, Pleisch G, Fraga-González G, Brem S. Development of Print-Speech Integration in the Brain of Beginning Readers With Varying Reading Skills. *Front Hum Neurosci.* 2020;14:289.

**86.** Ward D, Connally EL, Pliatsikas C, Bretherton-Furness J, Watkins KE. The neurological underpinnings of cluttering: Some initial findings. *J Fluency Disord.* Mar 2015;43:1-16.

**87.** Watkins KE, Smith SM, Davis S, Howell P. Structural and functional abnormalities of the motor system in developmental stuttering. *Brain.* Jan 2008;131(Pt 1):50-59.

**88.** Weiss Y, Katzir T, Bitan T. When transparency is opaque: Effects of diacritic marks and vowel letters on dyslexic Hebrew readers. *Cortex.* Oct 2016;83:145-159.

**89.** Wimmer H, Schurz M, Sturm D, et al. A dual-route perspective on poor reading in a regular orthography: An fMRI study. *CORTEX.* 2010 NOV-DEC 2010;46(10):1284-1298.

**90.** Yang J, Tan LH. Whole-Brain Functional Networks for Phonological and Orthographic Processing in Chinese Good and Poor Readers. *FRONTIERS IN PSYCHOLOGY.* 2020 JAN 14 2020;10.

**91.** Yang Y, Zuo Z, Tam F, et al. The brain basis of handwriting deficits in Chinese children with developmental dyslexia. *Dev Sci.* Mar 2022;25(2):e13161.

**92.** Yang Y, Yang YH, Li J, Xu M, Bi H-Y. An audiovisual integration deficit underlies reading failure in nontransparent writing systems: An fMRI study of Chinese children with dyslexia. *JOURNAL OF NEUROLINGUISTICS.* 2020 MAY 2020;54.

**eTable 3. Information of selected voxel-based morphometry studies**

| **Cit#** | **Study** | **Type of disorder** | **N_disorder** | **N_TD** | **Age (mean)** | **Age**  **(SD)** | **Subject type** | **Voxel level threshold** | **Cluster level threshold** | **N_hypo** | **N_hyper** |
| --- | --- | --- | --- | --- | --- | --- | --- | --- | --- | --- | --- |
|  |  |  |  |  |  |  |  |  |  |  |  |
| 1 | AdrianVentura et al., (2020) | DD ^a^ | 13 | 12 | 12 | 1.7 | C | NR | FWE *P<*0.05 | 9 | 0 |
| 2 | Beal et al., (2013) | PDS ^a^ | 11 | 11 | 2 | NR | C | *P<*0.001 | 37 voxels | 5 | 5 |
| 3 | Brambati et al., (2004) | DD ^a^ | 10 | 11 | 29 | NR | A | *P<*0.05 | NR | 9 | 0 |
| 4 | Brown et al., (2001) | DD ^a^ | 16 | 14 | 24 | 5 | A | *P<*0.05 | *P<*0.05 | 8 | 0 |
| 5 | Chang et al., (2008) | PDS ^a^ | 8 | 7 | 11 | 16.9 | C | *P<*0.001 | NR | 14 | 0 |
| 6 | Eckert et al., (2005) | DD ^a^ | 13 | 13 | 11 | 8.2 | C | *P<*0.001 | NR | 5 | 0 |
| 7 | Evans et al., (2013) | DD ^a^ | 14 | 14 | 42 | 9.7 | A | *P<*0.001 | FWE *P<*0.05 | 2 | 0 |
| 7 | Evans et al., (2013) | DD ^a^ | 13 | 13 | 31 | 10.7 | A | *P<*0.001 | FWE *P<*0.05 | 2 | 0 |
| 7 | Evans et al., (2013) | DD ^a^ | 15 | 15 | 9 | 1.7 | C | *P<*0.001 | FWE *P<*0.05 | 1 | 0 |
| 7 | Evans et al., (2013) | DD ^a^ | 17 | 17 | 10 | 2.6 | C | *P<*0.001 | FWE *P<*0.05 | 3 | 0 |
| 8 | Hoeft et al., (2007) | DD ^a^ | 19 | 19 | 14 | 2.2 | C | *P<*0.01 | *P<*0.01 | 6 | 0 |
| 9 | JaggerRickels et al., (2018) | DD ^a^ | 17 | 32 | 10 | NR | C | *P<*0.001 | NR | 12 | 0 |
| 10 | Jednorg et al., (2013) | DD ^a^ | 46 | 35 | 10 | 10.6 | C | *P<*0.001 | *P<*0.05 | 1 | 2 |
| 11 | Jednorg et al., (2015) | DD ^a^ | 130 | 106 | 10 | NR | C | *P<*0.001 | 150 voxels | 1 | 0 |
| 12 | Kikuchi et al., (2011) | PDS ^m^ | 15 | 15 | 30 | 6.1 | A | *P<*0.001 | NR | 3 | 4 |
| 13 | Krafnick et al., (2014) | DD ^a^ | 15 | 15 | 10 | 2.1 | C | *P<*0.01 | FWE *P<*0.01 | 5 | 0 |
| 14 | Kronbichler et al., (2008) | DD ^a^ | 13 | 15 | 16 | 0.7 | A | *P<*0.005 | NR | 11 | 8 |
| 15 | Liu et al., (2013S) | DD ^m^ | 18 | 18 | 12 | 5.6 | C | *P<*0.001 | 196 voxels | 8 | 0 |
| 16 | Siok et al., (2008S) | DD ^m^ | 16 | 16 | 11 | NR | C | FWE *P<*0.05 | 50 voxels | 1 | 0 |
| 17 | Wang et al., (2018) | DD ^m^ | 27 | 19 | 11 | NR | C | *P<*0.001 | FWE *P<*0.05 | 2 | 1 |
| 18 | Xia et al., (2016) | DD ^m^ | 24 | 24 | 13 | NR | C | *P<*0.001 | FWE *P<*0.05 | 3 | 0 |
| 19 | Yang et al., (2016) | DD ^m^ | 9 | 14 | 12 | 0.9 | C | *P<*0.005 | 40 voxels | 7 | 3 |

Notes:

^a^: alphabetic language disorder; ^m^: morpho-syllabic language disorder.

**Abbreviation:**

A: adults; C: children; DD: developmental dyslexia; FDR: false discovery rate; FWE: family wise error; PDS: persistent developmental stuttering; NR: not reported; N_disorder: number of subjects with DD or PDS; N_TD: number of typical developing subjects; N_hypo: number of hypoactivated foci; N_hyper: number of hyperactivated foci.

**1.** Adrian-Ventura J, Soriano-Ferrer M, Fuentes-Claramonte P, Morte-Soriano M, Antonia Parcet M, Avila C. Grey matter reduction in the occipitotemporal cortex in Spanish children with dyslexia: A voxel-based morphometry study. *JOURNAL OF NEUROLINGUISTICS.* 2020 FEB 2020;53.

**2.** Beal DS, Gracco VL, Brettschneider J, Kroll RM, De Nil LF. A voxel-based morphometry (VBM) analysis of regional grey and white matter volume abnormalities within the speech production network of children who stutter. *Cortex.* Sep 2013;49(8):2151-2161.

**3.** Brambati SM, Termine C, Ruffino M, et al. Regional reductions of gray matter volume in familial dyslexia. *Neurology.* Aug 24 2004;63(4):742-745.

**4.** Brown WE, Eliez S, Menon V, Rumsey JM, White CD, Reiss AL. Preliminary evidence of widespread morphological variations of the brain in dyslexia. *Neurology.* Mar 27 2001;56(6):781-783.

**5.** Chang SE, Erickson KI, Ambrose NG, Hasegawa-Johnson MA, Ludlow CL. Brain anatomy differences in childhood stuttering. *Neuroimage.* Feb 1 2008;39(3):1333-1344.

**6.** Eckert MA, Leonard CM, Wilke M, et al. Anatomical signatures of dyslexia in children: unique information from manual and voxel based morphometry brain measures. *Cortex.* Jun 2005;41(3):304-315.

**7.** Evans TM, Flowers DL, Napoliello EM, Eden GF. Sex-specific gray matter volume differences in females with developmental dyslexia. *Brain Struct Funct.* May 2014;219(3):1041-1054.

**8.** Hoeft F, Meyler A, Hernandez A, et al. Functional and morphometric brain dissociation between dyslexia and reading ability. *Proc Natl Acad Sci U S A.* Mar 6 2007;104(10):4234-4239.

**9.** Jagger-Rickels AC, Kibby MY, Constance JM. Global gray matter morphometry differences between children with reading disability, ADHD, and comorbid reading disability/ADHD. *Brain Lang.* Oct 2018;185:54-66.

**10.** Jednoróg K, Gawron N, Marchewka A, Heim S, Grabowska A. Cognitive subtypes of dyslexia are characterized by distinct patterns of grey matter volume. *Brain Struct Funct.* Sep 2014;219(5):1697-1707.

**11.** Jednoróg K, Marchewka A, Altarelli I, et al. How reliable are gray matter disruptions in specific reading disability across multiple countries and languages? Insights from a large-scale voxel-based morphometry study. *Hum Brain Mapp.* May 2015;36(5):1741-1754.

**12.** Kikuchi Y, Ogata K, Umesaki T, et al. Spatiotemporal signatures of an abnormal auditory system in stuttering. *Neuroimage.* Apr 1 2011;55(3):891-899.

**13.** Krafnick AJ, Flowers DL, Luetje MM, Napoliello EM, Eden GF. An investigation into the origin of anatomical differences in dyslexia. *J Neurosci.* Jan 15 2014;34(3):901-908.

**14.** Kronbichler M, Wimmer H, Staffen W, Hutzler F, Mair A, Ladurner G. Developmental dyslexia: gray matter abnormalities in the occipitotemporal cortex. *Hum Brain Mapp.* May 2008;29(5):613-625.

**15.** Liu L, You W, Wang W, Guo X, Peng D, Booth J. Altered brain structure in Chinese dyslexic children. *Neuropsychologia.* Jun 2013;51(7):1169-1176.

**16.** Siok WT, Niu Z, Jin Z, Perfetti CA, Tan LH. A structural-functional basis for dyslexia in the cortex of Chinese readers. *Proc Natl Acad Sci U S A.* Apr 8 2008;105(14):5561-5566.

**17.** Wang Z, Yan X, Liu Y, Spray GJ, Deng Y, Cao F. Structural and functional abnormality of the putamen in children with developmental dyslexia. *Neuropsychologia.* Jul 2019;130:26-37.

**18.** Xia Z, Hoeft F, Zhang L, Shu H. Neuroanatomical anomalies of dyslexia: Disambiguating the effects of disorder, performance, and maturation. *Neuropsychologia.* Jan 29 2016;81:68-78.

**19.** Yang Y-H, Yang Y, Chen B-G, Zhang Y-W, Bi H-Y. Anomalous Cerebellar Anatomy in Chinese Children with Dyslexia. *FRONTIERS IN PSYCHOLOGY.* 2016 MAR 18 2016;7.

**eTable 4. Regional activation abnormalities associated with the disorder group under alphabetic languages**

| Cluster | Voxels | Local peak | X | Y | Z | SDM-*Z* | BA |
| --- | --- | --- | --- | --- | --- | --- | --- |
| *Hypoactivation* |  |  |  |  |  |  |  |
| Left SMG | 1045 | Left SMG | -58 | -48 | 30 | -3.79 | 40 |
|  |  | Left MTG | -62 | -48 | 2 | -3.38 | 21 |
|  |  | Left STG | -56 | -48 | 16 | -3.34 | 22 |
|  |  | Left MTG | -56 | -54 | 4 | -3.33 | 21 |
|  |  | Left MTG | -58 | -52 | 14 | -3.33 | 21 |
|  |  | Left MTG | -60 | -46 | 8 | -3.32 | 22 |
|  |  | Left MTG | -56 | -54 | 0 | -3.29 | 21 |
|  |  | Left MTG | -56 | -46 | 6 | -3.28 | 22 |
|  |  | Left MTG | -52 | -52 | 20 | -3.27 | 22 |
|  |  | Left MTG | -60 | -52 | 10 | -3.25 | 21 |
|  |  | Left MTG | -54 | -58 | 6 | -2.94 | 37 |
| Left ITG | 854 | Left ITG | -46 | -50 | -20 | -4.52 | 20 |
|  |  | Left ITG | -46 | -56 | -14 | -4.50 | 37 |
|  |  | Left ITG | -50 | -56 | -16 | -4.37 | 37 |
|  |  |  |  |  |  |  |  |
| *Hyperactivation* |  |  |  |  |  |  |  |
| No foci |  |  |  |  |  |  |  |

Regions that survived with the statistical threshold set at *p* < 0.005, a cluster extent of 10 voxels and the peak SDM-*Z* > 1. Coordinates reported in Montreal Neurological Institute space.

**Abbreviation:**

BA: Broadman’s area; ITG: inferior temporal gyrus; MTG: middle temporal gyrus; SMG: supramarginal gyrus; STG: superior temporal gyrus.

**eTable 5. Meta-analytic connectivity modeling for the left inferior temporal gyrus**

| **Lobe** | **Region** | **BA** | | **X** | **Y** | **Z** | **ALE** |
| --- | --- | --- | --- | --- | --- | --- | --- |
| temporal | fusiform gyrus | 37 | | -46 | -56 | -16 | 0.45 |
|  | fusiform gyrus | 37 | | 46 | -56 | -16 | 0.11 |
|  | middle temporal gyrus | 37 | | -52 | -68 | 6 | 0.03 |
| frontal | superior frontal gyrus | | 6 | -2 | 16 | 50 | 0.13 |
|  | inferior frontal gyrus | | 9 | -44 | 8 | 26 | 0.10 |
|  | middle frontal gyrus | | 46 | -44 | 30 | 14 | 0.09 |
|  | middle frontal gyrus | | 6 | -40 | 0 | 44 | 0.07 |
|  | inferior frontal gyrus | | 47 | -42 | 26 | -6 | 0.06 |
|  | middle frontal gyrus | | 9 | 44 | 32 | 26 | 0.06 |
|  | middle frontal gyrus | | 46 | 50 | 30 | 20 | 0.05 |
|  | middle frontal gyrus | | 46 | 50 | 34 | 12 | 0.05 |
|  | inferior frontal gyrus | | 9 | 48 | 12 | 22 | 0.05 |
|  | inferior frontal gyrus | | 9 | 50 | 8 | 30 | 0.05 |
|  | inferior frontal gyrus | | 47 | 36 | 32 | -12 | 0.04 |
|  | middle frontal gyrus | | 6 | -28 | -2 | 56 | 0.04 |
| parietal | inferior parietal lobule | | 40 | -34 | -48 | 46 | 0.08 |
|  | superior parietal lobule | | 7 | 32 | -54 | 46 | 0.08 |
|  | superior parietal lobule | | 7 | -28 | -60 | 50 | 0.08 |
|  | inferior parietal lobule | | 40 | -42 | -42 | 44 | 0.06 |
|  | inferior parietal lobule | | 40 | 44 | -44 | 46 | 0.05 |
|  | precuneus | | 19 | -24 | -76 | 40 | 0.04 |
|  | precuneus | | 7 | 26 | -62 | 58 | 0.04 |
|  | inferior parietal lobule | | 40 | -52 | -30 | 38 | 0.04 |
|  | supramarginal gyrus | | 40 | 48 | -40 | 36 | 0.03 |
| occipital | inferior occipital gyrus | | 19 | 40 | -82 | -6 | 0.07 |
|  | fusiform gyrus | | 19 | -40 | -84 | -10 | 0.06 |
|  | middle occipital gyrus | | NA | -30 | -92 | 4 | 0.05 |
|  | precuneus | | 31 | -26 | -78 | 26 | 0.05 |
|  | fusiform gyrus | | 18 | 24 | -94 | -8 | 0.04 |
| sub-lobar | insula | | 13 | -34 | 24 | 0 | 0.07 |
|  | insula | | 13 | 34 | 24 | 4 | 0.06 |
|  | insula | | 13 | 40 | 24 | -2 | 0.05 |
|  | insula | | 13 | -46 | 8 | 2 | 0.03 |
| anterior | culmen | | NA | 28 | -56 | -20 | 0.04 |
|  | culmen | | NA | 32 | -64 | -26 | 0.04 |

Regions that survived with the *p* = 0.05 with 1000 permutations, and a cluster-level family wise error (*c*FWE) at voxel-level *p* < 0.001. Coordinates reported in Montreal Neurological Institute space. **Abbreviation:**

BA: Broadman’s area; NA: not applicable.

**eTable 6. Meta-analytic connectivity modeling for the left middle temporal gyrus**

| **Lobe** | **Region** | **BA** | **X** | **Y** | **Z** | **ALE** |
| --- | --- | --- | --- | --- | --- | --- |
| temporal | inferior temporal gyrus | 37 | -56 | -54 | 0 | 0.25 |
|  | middle temporal gyrus | 37 | 58 | -54 | 4 | 0.03 |
| sub-lobar | insula | 13 | -34 | 22 | 0 | 0.04 |
| frontal | precentral gyrus | 44 | -44 | 18 | 4 | 0.03 |
|  | precentral gyrus | 6 | -48 | -2 | 42 | 0.03 |
|  | precentral gyrus | 4 | -50 | -2 | 46 | 0.03 |
|  | middle frontal gyrus | 46 | -46 | 22 | 18 | 0.04 |
|  | middle frontal gyrus | 6 | -42 | 4 | 48 | 0.03 |
|  | inferior frontal gyrus | 44 | -52 | 10 | 16 | 0.05 |
|  | inferior frontal gyrus | 9 | -52 | 10 | 32 | 0.04 |
|  | inferior frontal gyrus | 45 | -52 | 24 | -2 | 0.02 |
|  | inferior frontal gyrus | 47 | -34 | 34 | -8 | 0.02 |
| occipital | middle occipital gyrus | 19 | 52 | -54 | 0 | 0.03 |
|  | inferior temporal gyrus | 37 | -52 | -70 | 6 | 0.02 |

Regions that survived with the *p* = 0.05 with 1000 permutations, and a cluster-level family wise error (cFWE) at voxel-level *p* < 0.001. Coordinates reported in Montreal Neurological Institute space. **Abbreviation:**

BA: Broadman’s area.

**eTable 7. Meta-analytic connectivity modeling for the left inferior parietal gyrus**

| **Lobe** | **Region** | **BA** | **X** | **Y** | **Z** | **ALE** |
| --- | --- | --- | --- | --- | --- | --- |
| parietal | supramarginal gyrus | 40 | -56 | -50 | 38 | 0.24 |
|  | supramarginal gyrus | 40 | 58 | -44 | 36 | 0.05 |
| sub-lobar | insula | NA | 38 | 22 | -4 | 0.04 |
|  | insula | NA | -40 | 18 | -6 | 0.04 |
|  | insula | 13 | 54 | -38 | 26 | 0.03 |
|  | insula | 13 | -32 | 24 | 0 | 0.03 |
|  | insula | 13 | -42 | 20 | 4 | 0.03 |
| temporal | superior temporal gyrus | 13 | 64 | -46 | 20 | 0.03 |
|  | superior temporal gyrus | 39 | 60 | -54 | 24 | 0.02 |
|  | superior temporal gyrus | 22 | -46 | -52 | 20 | 0.02 |
| frontal | precentral gyrus | 44 | 52 | 16 | 2 | 0.03 |
|  | medial frontal gyrus | 6 | -2 | 20 | 46 | 0.03 |
| limbic | cingulate gyrus | 32 | 6 | 20 | 40 | 0.02 |
|  | cingulate gyrus | 32 | 10 | 22 | 32 | 0.02 |
| occipital | middle temporal gyrus | 19 | -46 | -64 | 20 | 0.02 |

Regions that survived with the *p* = 0.05 with 1000 permutations, and a cluster-level family wise error (cFWE) at voxel-level *p* < 0.001. Coordinates reported in Montreal Neurological Institute space. **Abbreviation:**

BA: Broadman’s area; NA: not applicable.

**eTable 8. Meta-analytic connectivity modeling for the left supramarginal gyrus**

| **Lobe** | **Region** | **BA** | **X** | **Y** | **Z** | **ALE** |
| --- | --- | --- | --- | --- | --- | --- |
| temporal | supramarginal gyrus | 40 | -56 | -50 | 30 | 0.25 |
|  | supramarginal gyrus | 40 | 54 | -50 | 30 | 0.05 |
|  | middle temporal gyrus | 21 | -58 | -38 | -4 | 0.03 |
| parietal | inferior parietal lobule | 40 | 60 | -44 | 26 | 0.04 |
|  | inferior parietal lobule | 40 | 54 | -44 | 44 | 0.03 |
|  | precuneus | 7 | 8 | -54 | 36 | 0.03 |
|  | inferior parietal lobule | 40 | 38 | -54 | 40 | 0.02 |
| sub-lobar | insula | 13 | -36 | 18 | 0 | 0.04 |
|  | caudate | NA | -12 | 6 | 12 | 0.04 |
|  | claustrum | NA | 36 | 20 | 0 | 0.03 |
|  | insula | 13 | 48 | 18 | -2 | 0.03 |
| frontal | medial frontal gyrus | 9 | -44 | 22 | 32 | 0.03 |
|  | medial frontal gyrus | 6 | 4 | 4 | 56 | 0.03 |
|  | inferior frontal gyrus | NA | -52 | 20 | -2 | 0.02 |
|  | medial frontal gyrus | 9 | -44 | 16 | 28 | 0.02 |
|  | superior frontal gyrus | 6 | 4 | 14 | 58 | 0.02 |
| limbic | cingulate gyrus | 31 | -2 | -46 | 32 | 0.03 |
| occipital | middle temporal gyrus | 19 | -50 | -64 | 20 | 0.02 |

Regions that survived with the *p* = 0.05 with 1000 permutations, and a cluster-level family wise error (cFWE) at voxel-level *p* < 0.001. Coordinates reported in Montreal Neurological Institute space. **Abbreviation:**

BA: Broadman’s area; NA: not applicable.

**eFigure 1. Funnel plots**


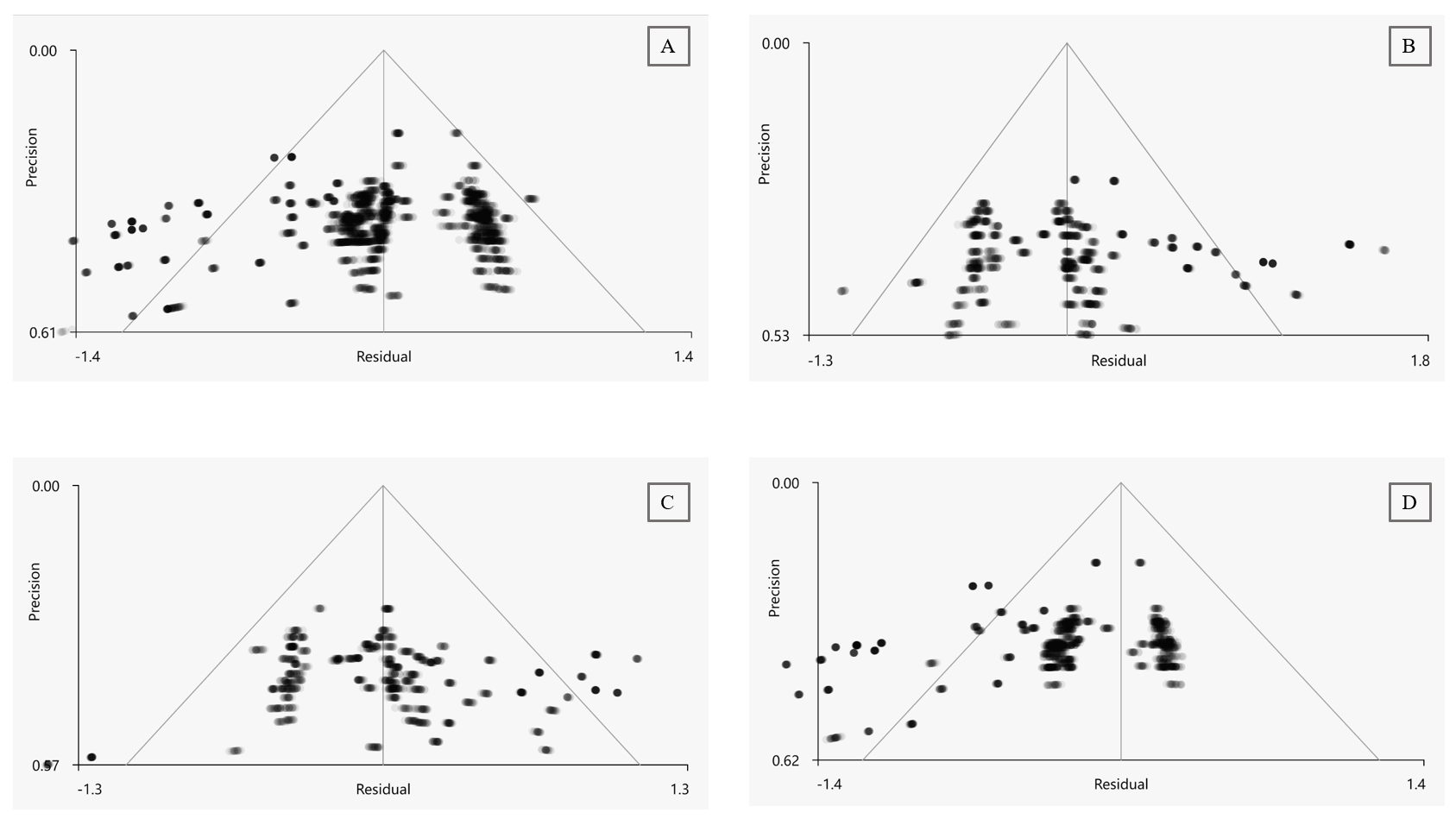


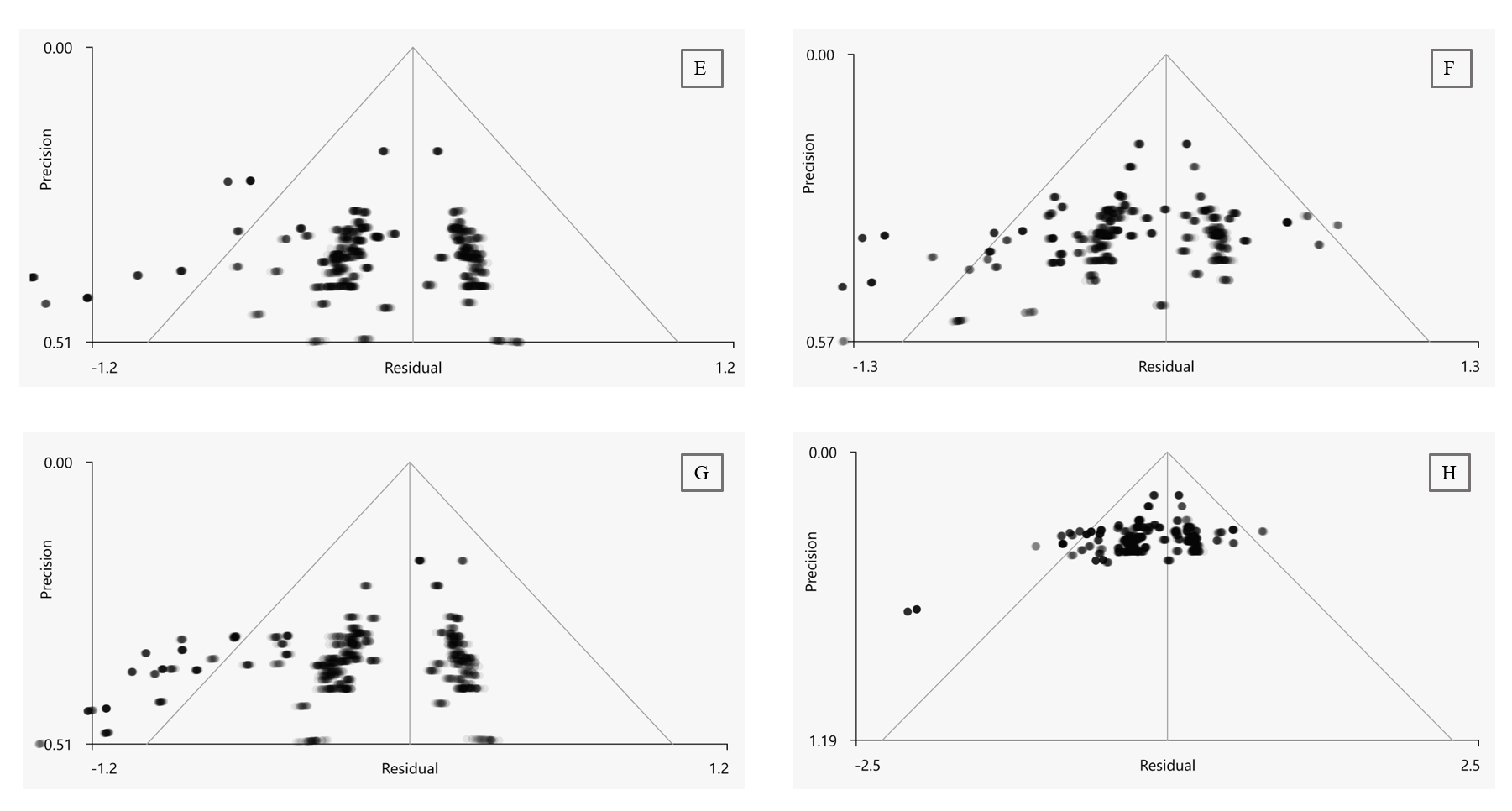


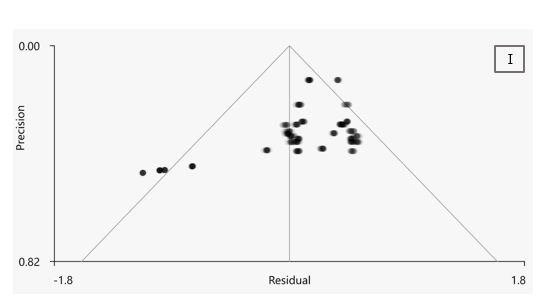


A. The symmetric funnel plot for the left inferior temporal gyrus (peak: -48, 56, -16) of the general functional meta-analysis across all DD and PDS. B and C. The symmetric funnel plots for the right precentral gyrus (peak: 54, 2, 26) and left median cingulate cortex (peak: -4, 20, 38) respectively of the functional meta-analysis in adults with DD or PDS. D, E and F. The symmetric funnel plots for the left inferior temporal gyrus (peak: -50, -56, -16), left middle temporal gyrus (peak: -56, -54, 22), and left inferior frontal gyrus (peak: -52, 10, 8) of the functional meta-analysis in children with DD or PDS. G and H. The asymmetric funnel plots for the left inferior parietal gyrus (peak: -46, -42, 46) and left precentral gyrus (peak: -50, 12, 30) of the functional meta-analysis in children with DD or PDS, indicating that results are driven by a small subset of studies or by studies with a small sample size. I. The symmetric funnel plot for the left inferior frontal gyrus (peak: -46, 20, 0) of the structural meta-analysis in children with DD or PDS. Coordinates reported in Montreal Neurological Institute space.

**eFigure 2. Regional activation abnormalities associated with the disorder group under alphabetic languages**


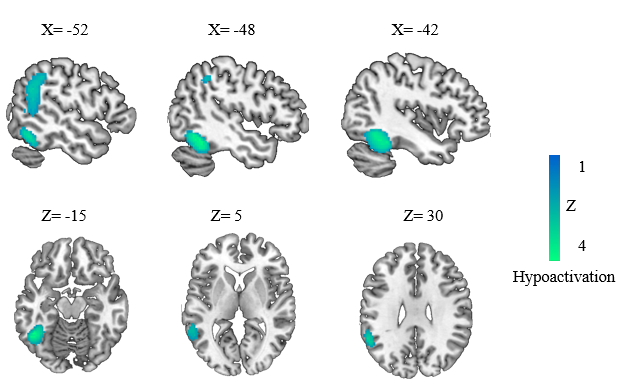


**eFigure 3. Results of the behavioral domain meta-analysis**


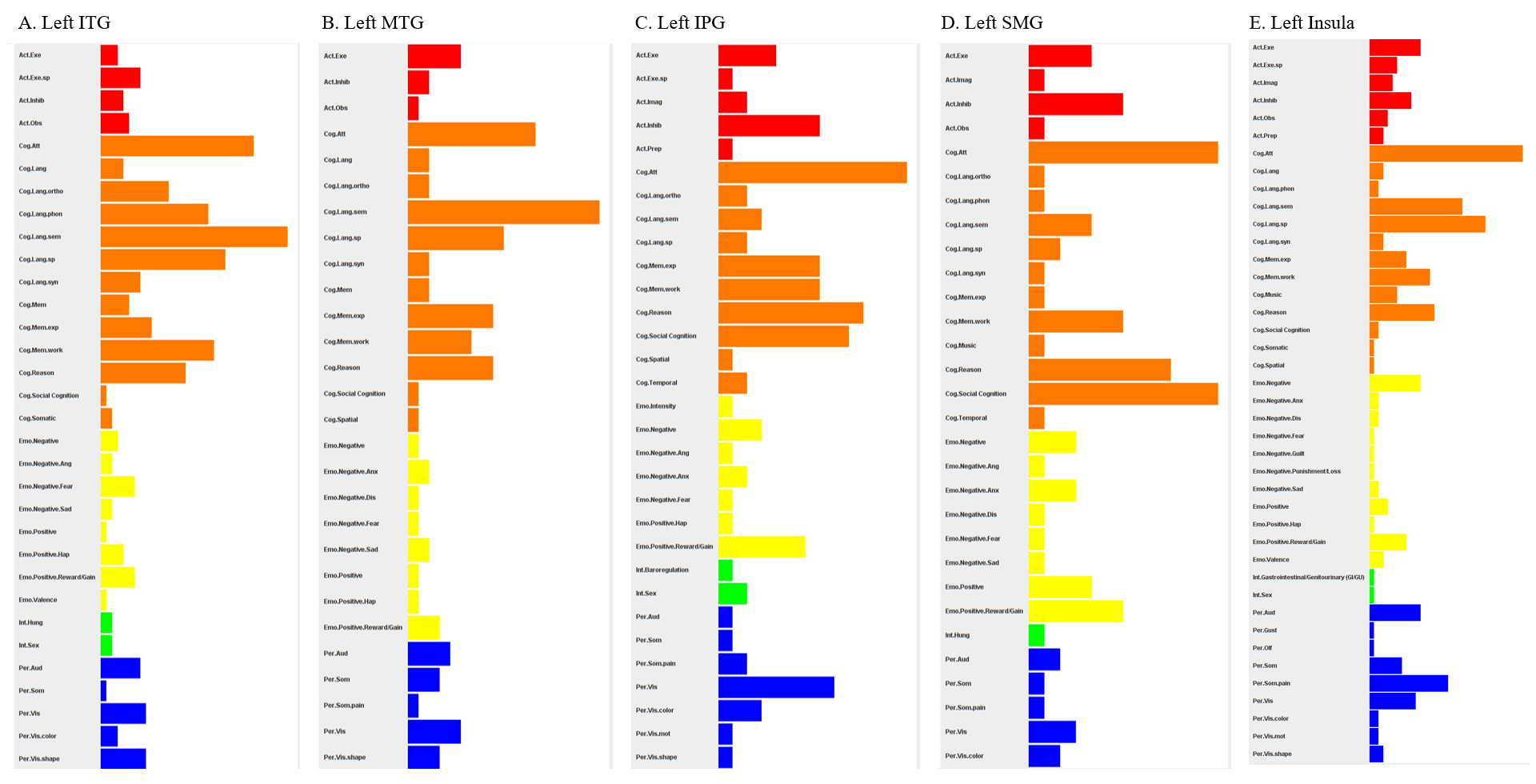


**Abbreviation:**

IPG: inferior parietal gyrus; ITG: inferior temporal gyrus; MTG: middle temporal gyrus; SMG: supramarginal gyrus.
